# Evolutionary Epigenetic Analysis of Oxidative Balance Score-Associated DNA Methylation Sites Across Mammals

**DOI:** 10.3390/ijms27198772

**Published:** 2026-09-30

**Authors:** Sun-Young Kang, Jeong-Soo Gim, Kyung-Wan Baek, Jeong-An Gim

**Affiliations:** 1 Institute for Molecular Metabolism Innovation, Soonchunhyang University, Asan 31538, Republic of Korea; 2 Department of Pet Health Care, Busan Health University, Busan 49318, Republic of Korea; 3 Research Institute of Pharmaceutical Sciences, Gyeongsang National University, Jinju 52828, Republic of Korea; 4 Department of Medical Science, Soonchunhyang University, Asan 31538, Republic of Korea

**Keywords:** Oxidative Balance Score (OBS), DNA methylation, comparative epigenetics, evolutionary decay, mammalian methylation consortium

## Abstract

Oxidative Balance Score (OBS) summarizes diet- and lifestyle-related exposures relevant to redox homeostasis, but the evolutionary behavior of OBS-associated epigenetic markers remains unclear. In this study, we conducted a covariate-adjusted epigenome-wide association study (EWAS) in Korean Genome and Epidemiology Study (KoGES) cohorts, adjusting for OBS and cell-type proportions. We identified 26 robust OBS-associated CpG sites. We then mapped these loci to the Mammalian Methylation Consortium dataset (GSE223748) and examined age-related methylation dynamics across nine mammalian species, using chronological age as a proxy for cumulative oxidative burden. Cross-species analyses showed that human correlation patterns were only partially preserved, with greater concordance observed in primates than in several non-primate species. Furthermore, 1000-iteration bootstrap resampling and null-model sensitivity analyses (Robinson-Foulds distance) demonstrated that the interspecies clustering topology of these 26 CpGs is statistically robust and distinct from random genomic noise. These findings suggest partial evolutionary preservation of age-related methylation behavior at human OBS-associated loci, providing a comparative framework for redox-related epigenetic dynamics.

## 1. Introduction

Oxidative stress, characterized by an imbalance between the production of reactive oxygen species (ROS) and the biological system’s capacity to detoxify reactive intermediates, is a hallmark of aging and a central pathogenic mechanism in diverse human diseases, including cardiovascular disorders, neurodegeneration, and metabolic syndromes [1,2]. The “Redox Hypothesis” suggests that while low levels of ROS serve as critical signaling molecules for cellular homeostasis, chronic supraphysiological levels lead to macromolecular damage, inflammation, and cellular senescence [3].

To assess this complex physiological state in clinical and epidemiological settings, the “Oxidative Balance Score (OBS)” has emerged as a comprehensive tool. Unlike single biomarkers, such as serum Vitamin C or lipid peroxidation levels, OBS integrates multiple dietary (pro- and antioxidants) and lifestyle factors (smoking or physical activity) to quantify an individual’s overall redox status [4]. Higher OBS is consistently associated with reduced all-cause mortality, longer telomeres, and lower cancer risk, highlighting its utility as a proxy for systemic oxidative health [5].

However, the molecular mechanisms translating these environmental oxidative exposures into long-term physiological outcomes remain incompletely understood. Epigenetic modifications, particularly DNA methylation, act as an interface between the environment and the genome [6]. Oxidative stress can directly reshape the DNA methylation landscape by inhibiting the activity of DNA methyltransferases (DNMTs) or by promoting the recruitment of Ten-Eleven Translocation (TET) enzymes to damage sites, creating “epigenetic scars” that persist over time [7,8]. Indeed, specific CpG sites have been identified as biomarkers of cumulative oxidative damage [9], and advanced epigenetic clocks like GrimAge incorporate smoking-related methylation metrics to predict lifespan with high accuracy [10].

Despite these advances, a fundamental biological question remains. Are the epigenetic mechanisms regulating redox balance unique to humans, or are they evolutionarily conserved traits essential for mammalian survival? The evolutionary theory of aging, particularly the “Disposable Soma” hypothesis, suggests that long-lived species invest more energy in somatic maintenance and repair mechanisms compared to short-lived species [11]. Given the exceptional longevity of humans within the mammalian class, we hypothesized that human-specific epigenetic regulation of oxidative stress might be linked to this extended lifespan, contrasting with the mechanisms found in shorter-lived rodents.

Comparative epigenetics offers a powerful lens to address this. By analyzing DNA methylation across diverse mammalian species, we can distinguish between potentially conserved age-related epigenetic trajectories and those that are species-specific adaptations [12,13]. This distinction is crucial for translational medicine, as it clarifies the limitations of animal models in studying human redox-driven diseases and identifies robust therapeutic targets that exhibit consistent cross-species patterns [14].

In this study, we performed a multi-stage evolutionary epigenetic analysis. First, we identified robust OBS-associated CpG sites in a large-scale human cohort (Korean Genome and Epidemiology Study, KoGES) [15]. Second, leveraging the Mammalian Methylation Consortium dataset [16], we traced the evolutionary trajectory of these markers across nine mammalian species. We specifically aimed to (1) determine whether OBS-related epigenetic signatures are conserved in other primates and mammals, (2) quantify the “Phylo-Correlation Decay” to understand the divergence of redox regulation, and (3) explore sex-specific evolutionary patterns that might explain the well-known sexual dimorphism in lifespan and oxidative stress resilience. Due to the practical challenges in obtaining species-specific OBS for each individual group, age was employed as a proxy based on the Free Radical Theory. Our findings provide new insights into the evolutionary origins of human redox regulation and highlight the potential of these conserved CpGs as targets for antioxidant-based interventions.

## 2. Results

### 2.1. Baseline Characteristics and Identification of OBS-Associated CpG Sites in the KoGES Cohort

The baseline demographic and lifestyle characteristics of the participants from the Ansan-Ansung (ASAS) cohorts, stratified by sex, are summarized in Table 1. The distribution of OBS scores among the participants is shown in Figure S1. Furthermore, scatter plots illustrating the individual M-values versus OBS scores for all 26 identified CpGs are provided in Figure S2. To identify DNA methylation markers strictly associated with OBS, independent of chronological aging and blood cell composition, we applied a covariate-adjusted multiple linear regression model (limma) utilizing M-values. By adjusting for OBS and estimated cell-type proportions (CD8T, CD4T, NK, B cells, and Monocytes), we effectively removed potential confounding artifacts. Within the ASAS cohorts, we identified 26 highly robust OBS-associated CpG sites (12 in males, 14 in females) that satisfied the stringent false discovery rate (FDR < 0.05) and nominal *p*-value thresholds (*p* < 0.01) (Table 2).

Venn diagram analysis revealed no overlapping markers (0.0%) between the OBS-associated CpG sites identified in males (*n* = 12) and females (*n* = 14) (Figure S3A). Furthermore, a cross-sex evaluation, assessing the male-specific CpGs in the female cohort and vice versa, demonstrated that while methylation data were available for the majority of these loci (11 of 12 male-associated sites and 12 of 14 female-associated sites), heatmap and scatter plot analyses showed no clear directional consistency or significant correlations across sexes (Figure S3B–D). These findings suggest that the DNA methylation dynamics associated with OBS are highly independent and operate in a strictly sex-specific manner.

To evaluate the functional and clinical relevance of the 26 OBS-associated CpGs, we analyzed multi-omics data across 31 of The Cancer Genome Atlas (TCGA) cancer cohorts. Correlation analyses revealed strong inverse methylation–expression relationships, highlighted by a ubiquitous negative correlation for *ZNF549* across nearly all evaluated malignancies. Broad inverse correlations were also prominently observed for *IRS1*, *LAMC2*, *MPPED2*, *PRKCE*, and *EVX1* across various solid tumors (Table S1).

Furthermore, several loci exhibited significant molecular alterations linked to disease severity. Distinct log2 fold-change shifts in gene expression and DNA methylation were consistently observed when comparing deceased versus surviving patients (Figure S4A,B), as well as advanced (Stage III/IV) versus early (Stage I/II) tumor stages (Figure S4C,D).

**Figure 1:**
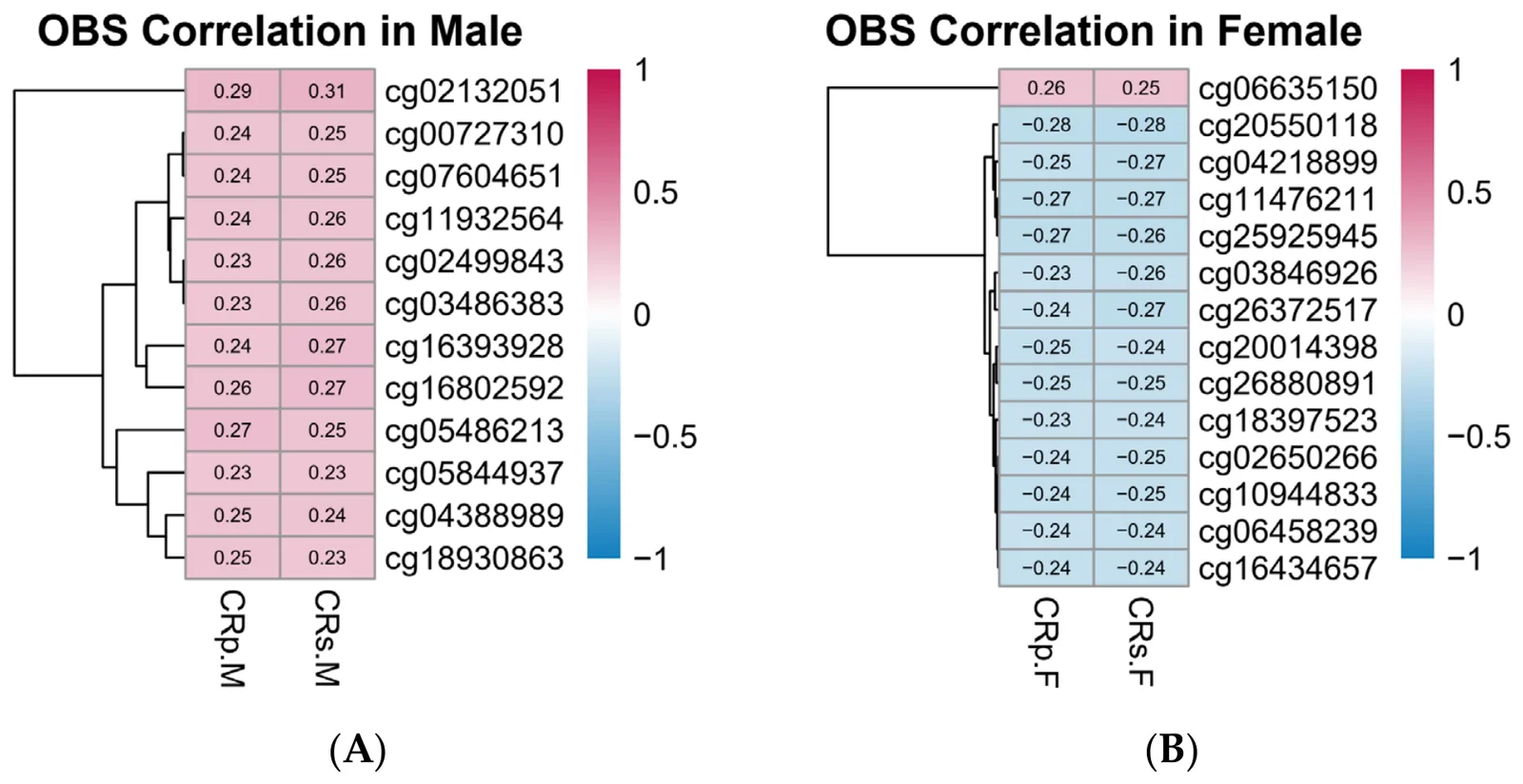
Sex-stratified heatmaps of OBS-associated CpG sites in the KoGES cohort. Heatmaps show the Pearson (r_p_) and Spearman (r_s_) correlation coefficients between OBS and DNA methylation beta values for the 26 CpG sites retained after dual-threshold filtering in the KoGES cohort (*n* = 12 for males, *n* = 14 for females). Panel (**A**) shows the male-specific CpG set, and Panel (**B**) shows the female-specific CpG set. Rows represent CpG sites, and columns represent correlation metrics. Red indicates positive correlation and blue indicates negative correlation. CpG sites were retained when they satisfied the predefined criteria of ∣r_p_∣ > 0.23 and ∣r_s_∣ > 0.23, and nominal *p* < 0.003 in the relevant sex-stratified analysis. Hierarchical clustering was applied to visualize similarity in correlation patterns across CpGs.

The selected CpGs mapped to a mixture of annotated and unannotated loci, with annotated sites including *IRS1*, *RGS6*, *PLXNA4*, *SLC7A5*, *C10orf140*, *LAMC2*, *MPPED2*, *ZNF549*, *FEZF1*, *NUP153*, *PRKCE*, *TNFRSF13C*, *PRDM16*, *LOC145845*, *EVX1*, *DSCAML1*, *PAX6*, *CRABP1*, *NCKAP1*, *TFAP2E*, and *ARIH1* (Table 2). Overall, the heatmaps indicated coherent correlation structure within each sex-specific CpG set rather than isolated single-site signals, suggesting that OBS-associated methylation changes were distributed across multiple loci (Figure 1).

To assess the potential biological relevance of the annotated subset, we mapped the corresponding host genes to Ingenuity Pathway Analysis (IPA). The resulting pathway assignments linked these genes to RAR activation, growth hormone signaling, transformation of prostate cancer cell lines, and serous adenocarcinoma (Table 3). Because these results were generated from in silico pathway mapping, they were interpreted as functional hypotheses rather than direct mechanistic validation.

### 2.2. Cross-Species Age-Related Correlations of Human OBS-Associated CpG Sites

**Figure 2:**
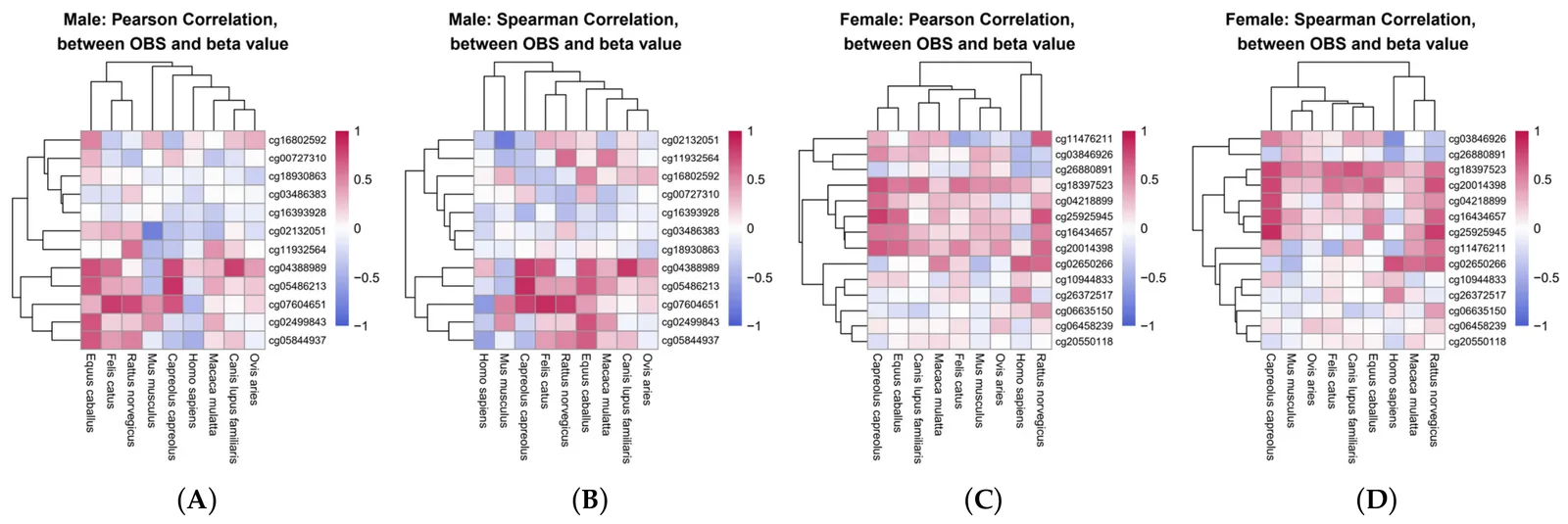
Cross-species heatmaps of age-related correlations for human OBS-associated CpG sites. Heatmaps display the Pearson and Spearman correlation coefficients between chronological age and DNA methylation beta values for the 26 human OBS-associated CpG sites across nine mammalian species. Panel (**A**) shows male Pearson correlations, Panel (**B**) male Spearman correlations, Panel (**C**) female Pearson correlations, and Panel (**D**) female Spearman correlations. Species included in the analysis were *Homo sapiens*, *Macaca mulatta*, *Mus musculus*, *Rattus norvegicus*, *Canis lupus familiaris*, *Felis catus*, *Ovis aries*, *Equus caballus*, and *Capreolus capreolus*. Red indicates positive correlation and blue indicates negative correlation. Because species-specific OBS was unavailable, chronological age was used as a proxy variable for cross-species comparison.

We next examined whether the 26 human OBS-associated CpG sites showed age-related methylation correlations across nine mammalian species using the Mammalian Methylation Consortium dataset (GSE223748). Because species-specific OBS was unavailable, chronological age was used as a proxy variable for cross-species comparison. Cross-species heatmaps of Pearson and Spearman coefficients showed that several CpGs retained non-zero age-related correlation patterns outside humans, although both the direction and magnitude of the correlations varied by species and sex (Figure 2; Table S2). Because the cross-species component relied on age rather than species-specific OBS, the term ‘conservation’ in this study refers to preservation of age-related correlation patterns at human OBS-associated CpGs, not direct conservation of the OBS phenotype itself.

**Figure 3:**
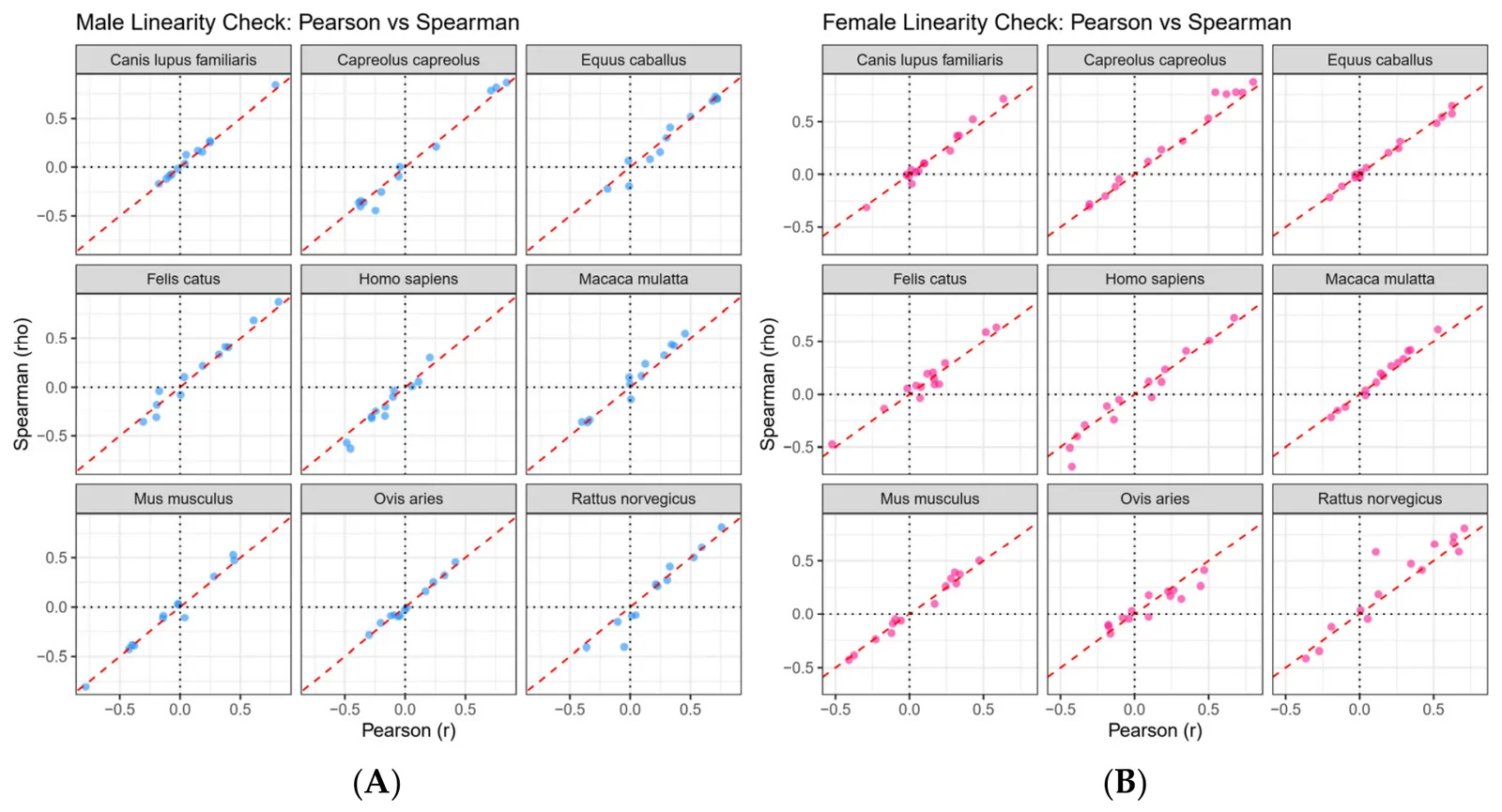
Concordance between Pearson and Spearman correlation estimates across species. Scatter plots compare Pearson and Spearman correlation coefficients calculated for the 26 OBS-associated CpG sites within each species. Panel (**A**) shows males and Panel (**B**) shows females. Each point represents one CpG site. The red dashed diagonal indicates the line of identity (y = x). Closer agreement between Pearson and Spearman coefficients indicates greater concordance between linear and rank-based association patterns, whereas wider dispersion indicates reduced concordance or increased heterogeneity across CpGs.

Pearson and Spearman coefficients were generally concordant within species, particularly in humans and rhesus macaques, where CpG-level estimates clustered relatively closely around the line of identity (Figure 3). In contrast, wider dispersion was observed in several non-primate species, indicating lower agreement between linear and rank-based association patterns and greater heterogeneity across CpGs.

### 2.3. Species-Level Variation in the Magnitude of Correlation

**Figure 4:**
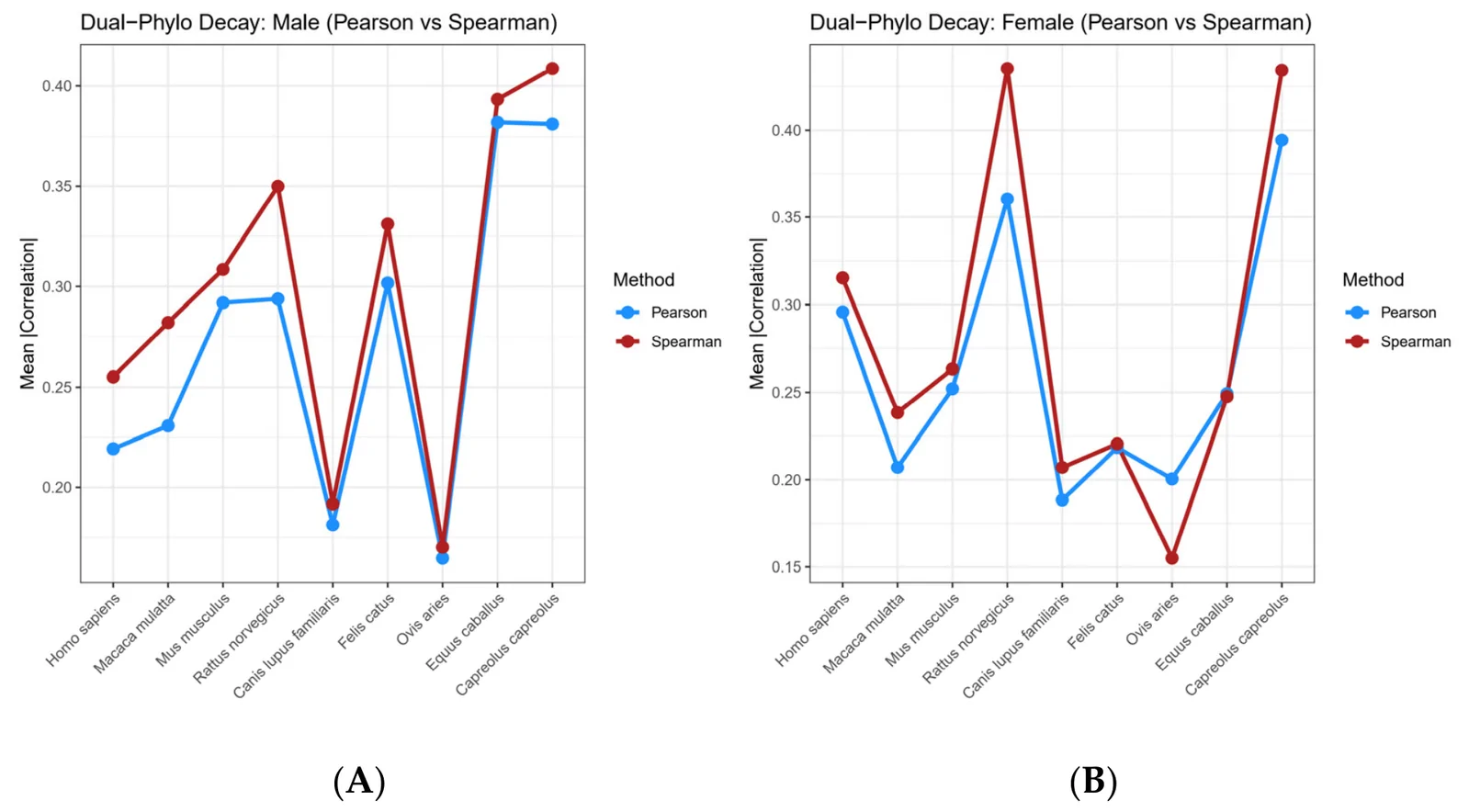
Mean absolute correlation strength across species for the OBS-associated CpG set. Line plots show the species-level mean absolute Pearson and Spearman correlation coefficients calculated from the 26 CpG sites. Panel (**A**) shows males and Panel (**B**) shows females. Blue lines indicate Pearson coefficients and red lines indicate Spearman coefficients. Species are displayed in the same order used in the cross-species analyses to facilitate comparison across figures. The plots summarize how the overall magnitude of age-related methylation correlation varies across mammalian species for the human OBS-associated CpG set.

To summarize the overall strength of age-related association for the 26 CpGs within each species, we calculated the mean absolute correlation coefficient across CpGs for both Pearson and Spearman metrics. This analysis showed that species-level correlation magnitude varied across mammals and that Pearson and Spearman trends were broadly similar within each sex (Figure 4).

**Figure 5:**
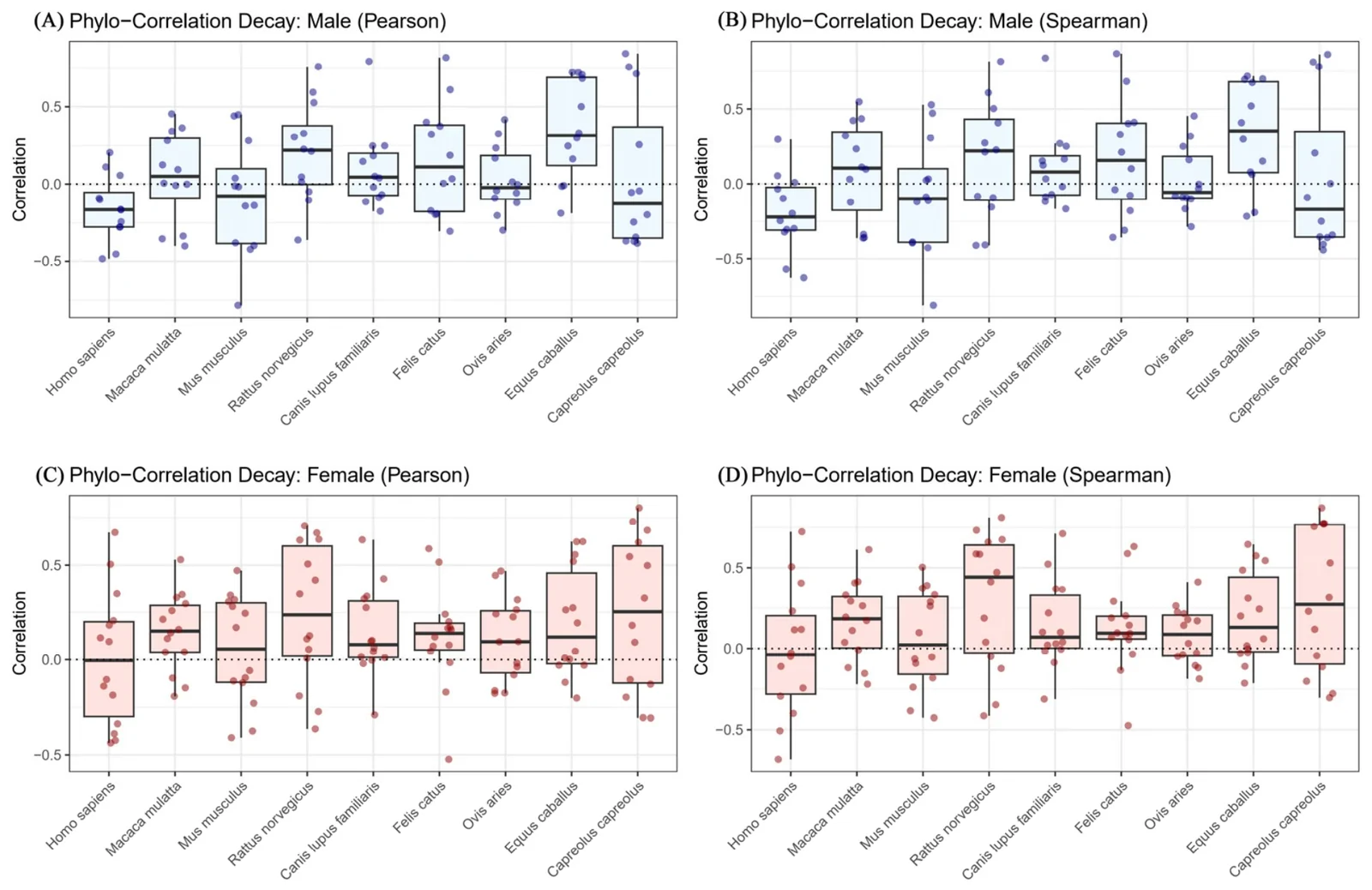
Distribution of cross-species correlation coefficients for the OBS-associated CpG set. Boxplots with overlaid jittered points show the distribution of CpG-level correlation coefficients across species. Panel (**A**) shows male Pearson correlations, Panel (**B**) male Spearman correlations, Panel (**C**) female Pearson correlations, and Panel (**D**) female Spearman correlations. Each point represents one CpG site. Distributions centered farther from zero indicate stronger age-related associations for the OBS-associated CpG set, whereas distributions centered nearer zero indicate weaker or less consistent associations across CpGs.

Boxplot-based summaries further show that the distribution of CpG-level correlation coefficients differed by species (Figure 5). In humans and rhesus macaques, several CpGs remained clearly separated from zero in both positive and negative directions, whereas in a number of non-primate species the distributions were more concentrated around zero, indicating weaker or less consistent age-related correlation across the prioritized CpG set. Female profiles showed a pattern broadly similar to that in males, although the spread of coefficients appeared more variable in some species.

### 2.4. Concordance and Divergence Relative to Human Correlation Patterns

**Figure 6:**
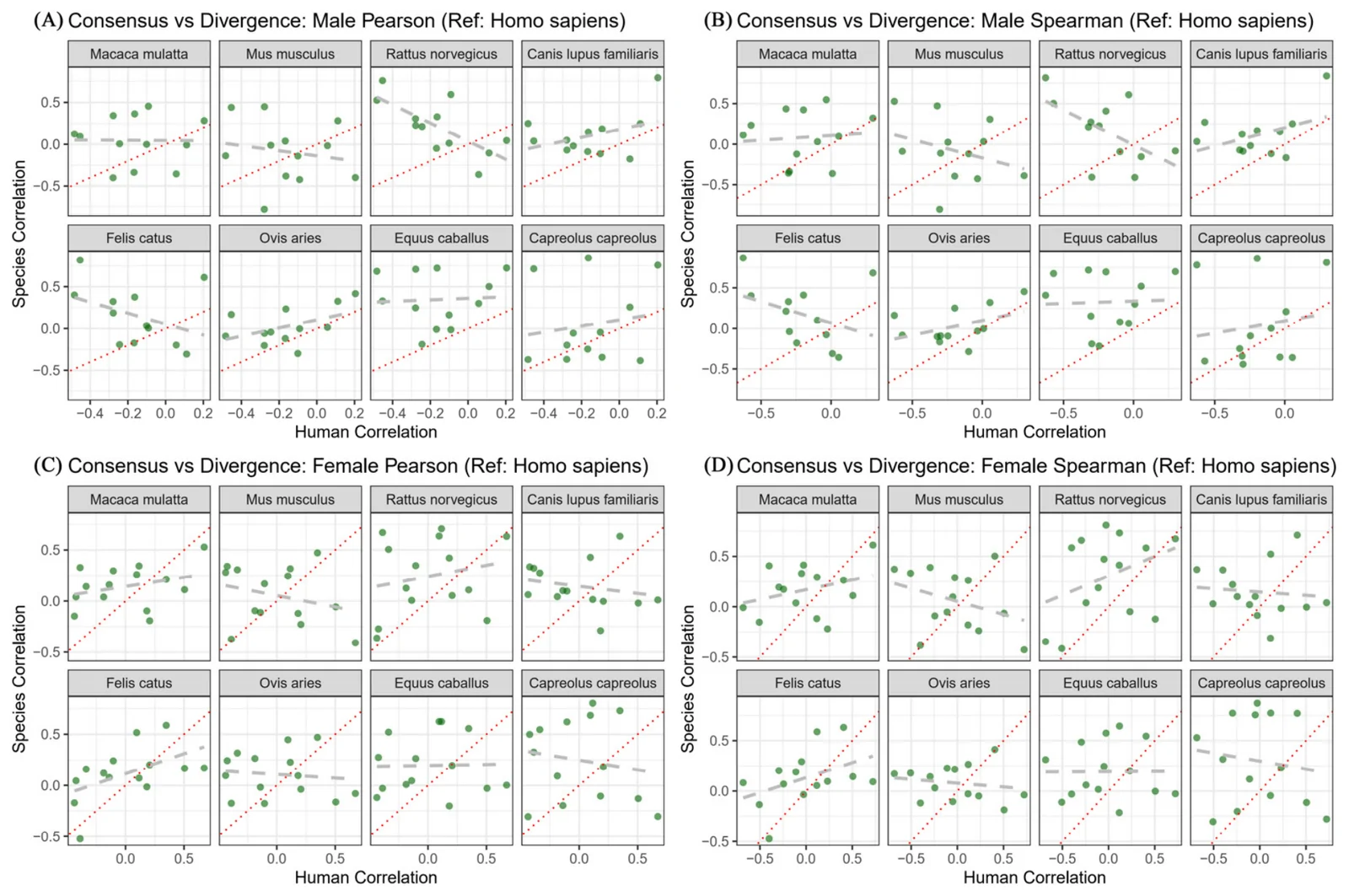
Concordance between human and non-human correlation patterns. Scatter plots compare species-specific correlation coefficients with the corresponding human correlation coefficients for the 26 OBS-associated CpG sites. Panel (**A**) shows male Pearson correlations, Panel (**B**) male Spearman correlations, Panel (**C**) female Pearson correlations, and Panel (**D**) female Spearman correlations. Each small panel corresponds to one non-human species. The red dashed diagonal indicates the line of identity. Gray regression lines summarize the degree of concordance between each species and humans, with steeper positive slopes indicating greater similarity in the CpG-level correlation pattern.

We then compared the CpG-level correlation profile of each non-human species with the corresponding human profile. Among the species examined, rhesus macaque showed the greatest visual concordance with humans across multiple analysis panels, whereas several non-primate species showed shallower or less consistent regression trends (Figure 6). These results suggest that the human correlation pattern is preserved to different degrees across mammals rather than being uniformly shared.

Importantly, concordance was not identical across all CpGs within a given species. Instead, the cross-species comparison indicated partial preservation of the human pattern at some loci together with substantial divergence at others, supporting a model of incomplete evolutionary conservation.

### 2.5. Multivariate Structure and Clustering of Species-Level Correlation Profiles

**Figure 7:**
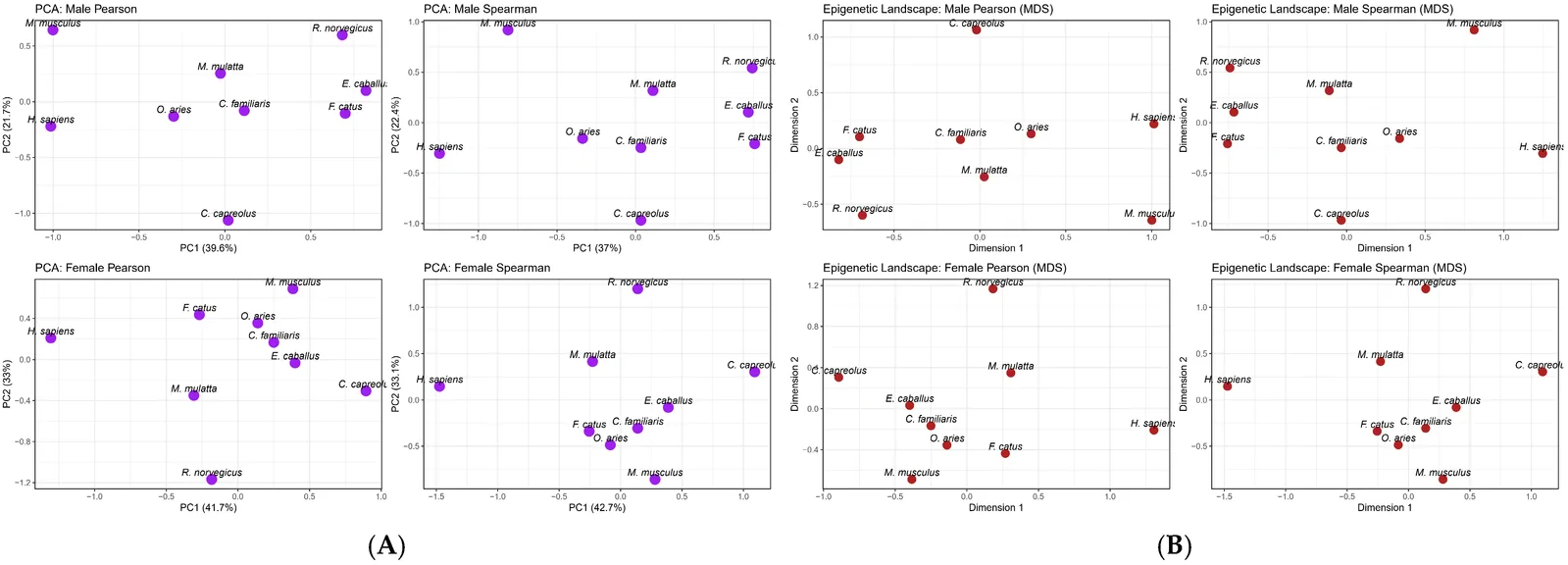
Multivariate representation of cross-species correlation profiles. Principal component analysis (PCA) and multidimensional scaling (MDS) were performed using species-by-CpG correlation matrices derived from the 26 OBS-associated CpG sites. Panel (**A**) shows PCA and Panel (**B**) shows MDS. Separate plots are provided for males and females and for Pearson and Spearman correlation matrices. These visualizations summarize the relative similarity of species-level epigenetic correlation profiles in a reduced two-dimensional space.

To assess the higher-order structure of these cross-species correlation patterns, we applied principal component analysis (PCA) and multidimensional scaling (MDS) to the species-level correlation matrices. In both analyses, species occupied partially separated positions in reduced-dimensional space, indicating that the correlation profiles of the 26 CpGs captured structured interspecies variation rather than random noise (Figure 7).

**Figure 8:**
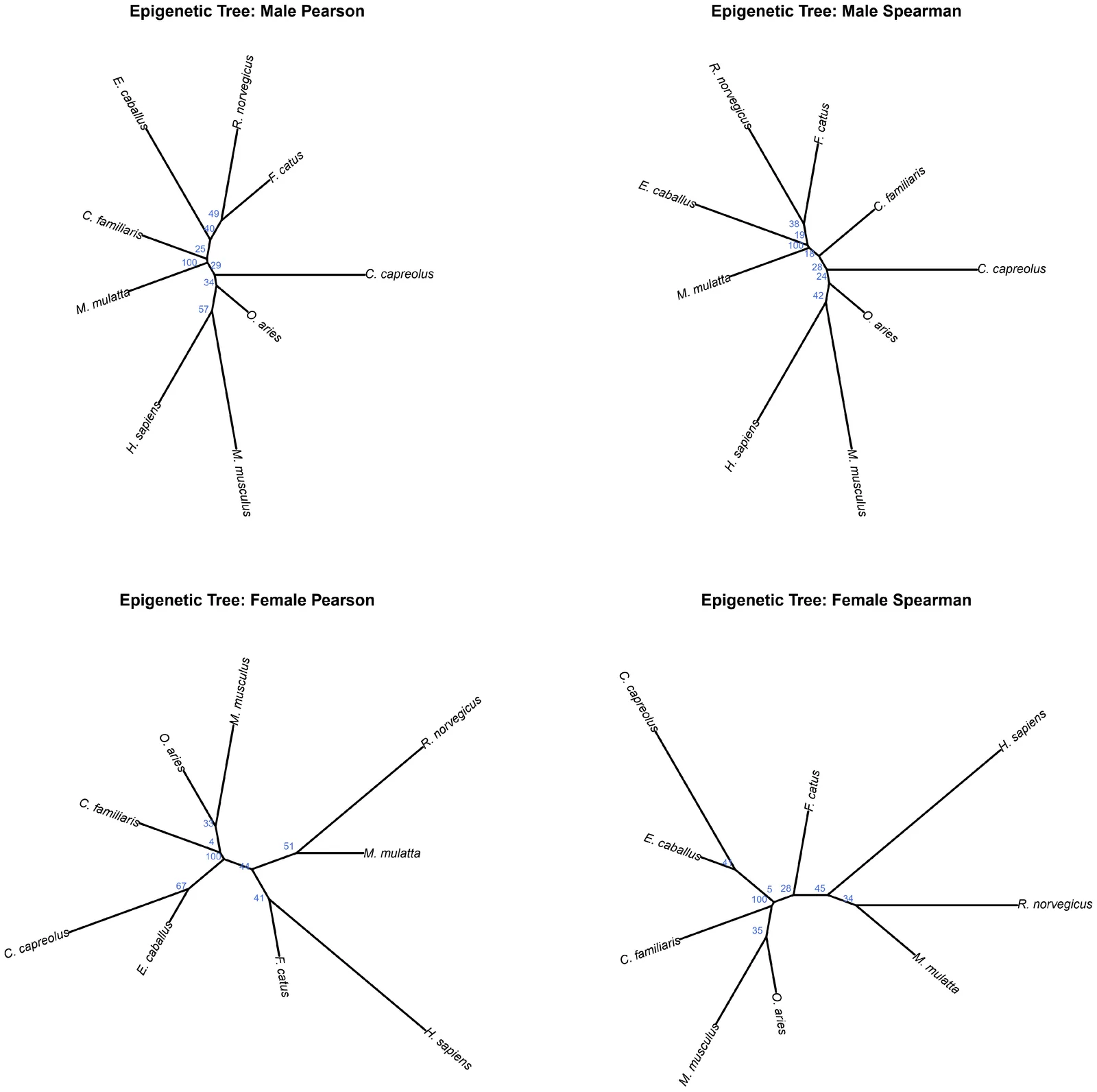
Neighbor-joining trees derived from cross-species epigenetic distance profiles with bootstrap support. Unrooted neighbor-joining (NJ) trees were constructed based on Euclidean distances calculated from the species-level correlation profiles of the 26 OBS-associated CpG sites. These trees represent descriptive similarity clustering within a reduced epigenetic feature space rather than true species-level evolutionary phylogenies. Separate trees are shown for males and females using both Pearson and Spearman correlation matrices. To evaluate the topological stability and robustness of the clustering, a 1000-iteration bootstrap resampling analysis was performed. The blue numbers located at the nodes indicate the bootstrap support values (expressed as percentages), representing the frequency with which a particular clade was recovered across the 1000 bootstrap replicates. Branch lengths reflect the relative epigenetic distance within the reduced CpG correlation space.

**Figure 9:**
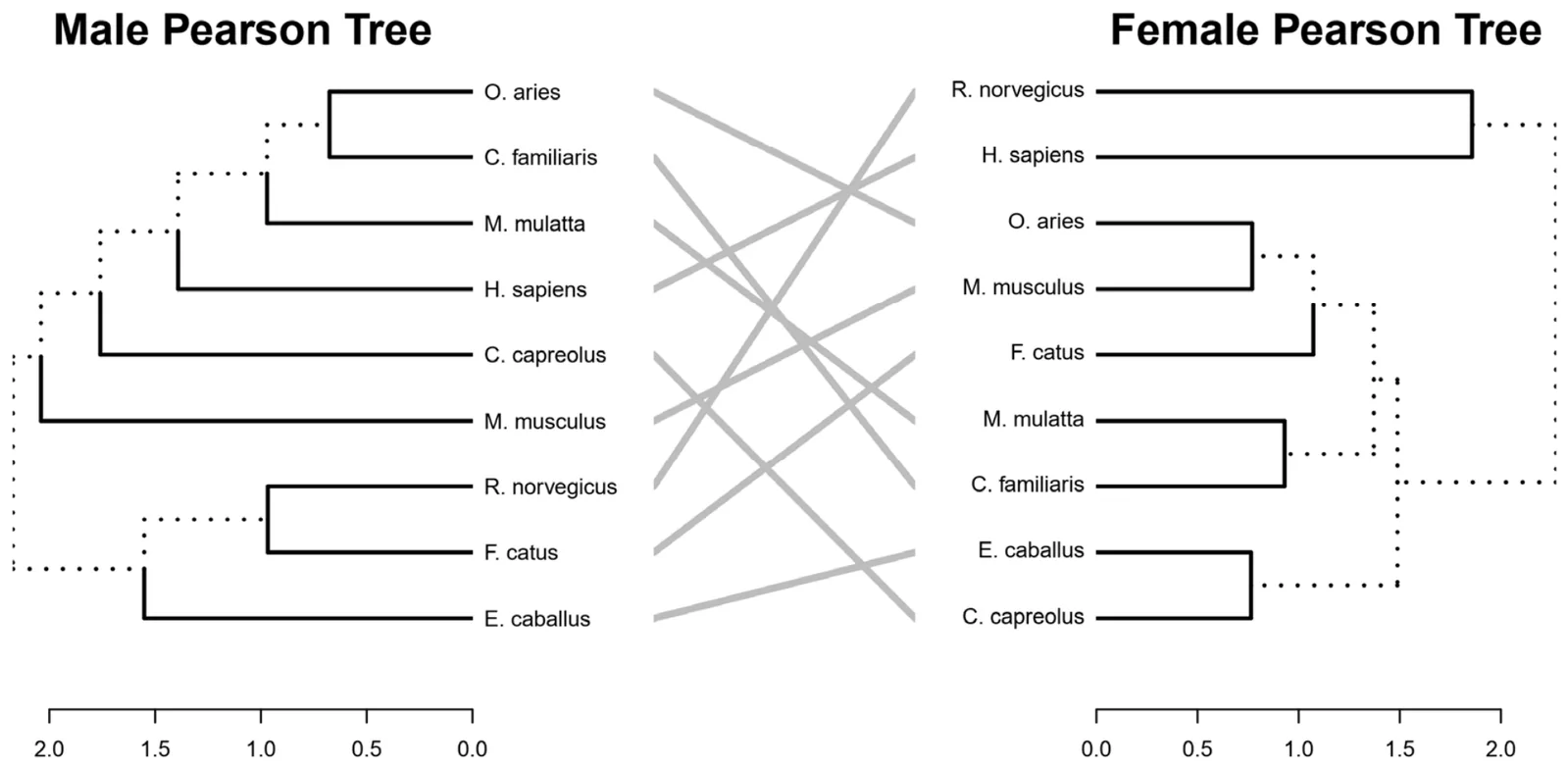
Tanglegram comparing male and female correlation-based dendrograms. The tanglegram compares dendrograms derived from male (**left**) and female (**right**) species-level correlation profiles for the OBS-associated CpG set. Solid branch lines represent the clustering structure within each sex-specific dendrogram. Solid lines crossing the center connect the same species between the male and female trees. Dotted lines indicate graphical branch extensions used to align the dendrograms and labels for visual comparison and do not represent additional evolutionary distance or statistical support. Greater crossing of the connecting lines indicates greater differences in species arrangement between the male and female clustering structures.

We further summarized these relationships by constructing neighbor-joining trees from Euclidean distances calculated on the correlation matrices. Although these trees were derived from a limited set of CpGs and should therefore be interpreted cautiously, they showed recognizable grouping structure across species (Figure 8). Given the limited number of CpGs used for distance calculation, the neighbor-joining trees are presented as descriptive summaries of similarity rather than as formal substitutes for genome-scale phylogenetic inference. Comparison of male and female clustering patterns using a tanglegram suggested that the broad organization was similar between sexes but not identical, indicating modest sex-related differences in the arrangement of species within the reduced epigenetic correlation space (Figure 9).

To rigorously address the potential noise and topological instability inherent in using a restricted feature space, we performed a 1000-iteration bootstrap resampling analysis on the Neighbor-Joining trees. The resulting bootstrap values supported the core branching structures, particularly those separating primate lineages from non-primate clades. Furthermore, to validate the biological specificity of the 26 OBS-associated CpGs, we conducted a null-model sensitivity analysis. We randomly sampled size-matched CpG sets (*n* = 12 for males, *n* = 14 for females) from the highly conserved 37,000 mammalian CpG pool 1000 times and calculated the Robinson–Foulds (RF) topological distance against our true OBS-CpG trees. The true topology was highly distinct from the random noise distributions (Figure S5), confirming that the interspecies clustering driven by these 26 loci captures a specific evolutionary trajectory related to redox homeostasis rather than random background sequence drift.

## 3. Discussion

In this study, we identified 26 CpG sites associated with the OBS in a human population-based cohort and examined whether these loci showed age-related methylation correlations across multiple mammalian species. Three principal findings emerged from the analysis. First, the OBS-associated CpG set identified in the KoGES cohort showed internally consistent correlation structure within sex-stratified human analyses. Second, these human-prioritized CpGs showed variable age-related correlation patterns across species, with relatively greater concordance in primates than in several non-primate mammals. Third, male and female species-level clustering structures were broadly similar but not identical, suggesting modest sex-related differences in the organization of these cross-species correlation profiles.

The cross-species results support the view that a subset of human OBS-associated methylation signals is only partially conserved across mammals. In particular, rhesus macaque showed the greatest overall similarity to humans in both the heatmap-based and regression-based comparisons, whereas several non-primate species showed weaker or less consistent concordance. This pattern is compatible with previous comparative methylation studies showing that age-related epigenetic architecture contains both conserved and lineage-dependent components across mammals [16,17,18,19]. Our findings extend this perspective by focusing specifically on CpG sites selected from a lifestyle-related oxidative balance phenotype in humans.

At the same time, the present results do not establish that oxidative balance itself is regulated by identical molecular mechanisms across species. In the human cohort, the CpGs were selected using OBS, whereas the comparative analysis relied on chronological age as a proxy because comparable species-specific OBS measures were unavailable. Accordingly, the cross-species component of this study should be interpreted as an analysis of age-related behavior at human OBS-associated CpGs rather than as direct evidence that OBS has the same biological meaning in all mammals [1,2,3]. This distinction is important for avoiding overinterpretation of partial correlation concordance as mechanistic conservation.

The pathway analysis provided additional biological context for the annotated subset of CpGs by linking their host genes to oxidative stress response, mitochondrial regulation, apoptosis-related signaling, and extracellular matrix remodeling. These pathway assignments are plausible in light of the known relationship between redox biology, cellular maintenance, and aging [7,8,13]. However, because the IPA results were derived from in silico mapping rather than perturbation-based experiments, they should be regarded as hypothesis-generating rather than confirmatory. Future work using targeted epigenetic editing, longitudinal intervention studies, or orthogonal molecular assays will be required to determine whether methylation changes at these loci contribute functionally to redox regulation.

The sex-stratified analyses also merit cautious consideration. Although the tanglegram suggested some differences between male and female clustering structures, the overall similarity between the two trees remained substantial. We therefore interpret the sex-related finding as evidence of modest variation in cross-species correlation architecture rather than as proof of distinct sex-specific evolutionary trajectories [20,21]. Additional formal testing, including quantitative comparison of tree similarity and replication in independent datasets, would be necessary to establish the robustness of this pattern.

These findings also have implications for model selection in comparative epigenetic research [16,19]. The weaker concordance observed in several non-primate species suggests that human OBS-associated CpGs cannot be assumed to behave uniformly across mammalian systems. This does not invalidate non-primate models, but it does indicate that the transferability of human-prioritized epigenetic markers should be evaluated empirically rather than presumed. In this context, primate similarity in the present study may be useful for prioritizing loci for follow-up validation.

Several limitations should be acknowledged. First, the cross-species analysis used age as a proxy for cumulative oxidative burden, which is biologically reasonable but not equivalent to direct measurement of species-specific OBS or oxidative exposure. Second, the analysis was based on a restricted panel of 26 CpG sites rather than genome-wide methylation profiles. Consequently, the resulting topologies do not represent true species-level phylogenetic trees, but should instead be interpreted as purely descriptive similarity clustering within a reduced feature space, which fundamentally limits the strength of formal phylogenetic interpretation. Third, although the custom mammalian methylation array was intrinsically designed to target highly conserved loci across mammalian species [16], we cannot entirely rule out the confounding effects of sequence drift or species-specific single-nucleotide polymorphisms (SNPs) within the probe flanking regions. Such localized genetic divergence could perturb probe hybridization efficiency and artificially depress cross-species DNA methylation beta values. This technical bias could potentially mimic or exaggerate the observed ‘phylo-correlation decay.’ Because rigorous sequence alignments and local genetic conservation checks for these specific 26 CpG probes across all nine species were not directly performed in this study, the interpretation of cross-species divergence must be made with this technical limitation in mind.

Fourth, the pathway analyses were based on host-gene annotation and knowledge-base inference and therefore cannot establish causality. Fifth, the sex-stratified comparative analysis relies on partially distinct CpG sets (12 in males, 14 in females), which limits the direct comparability of male and female evolutionary clustering topologies. Consequently, the observed differences between sexes in the dendrograms and tanglegrams should be interpreted as modest variations in correlation architecture rather than definitive proof of distinct sex-specific evolutionary trajectories. Furthermore, the lack of complete physiological and reproductive data (e.g., estrous cycles or hormonal variations) across the non-human species restricts our ability to fully contextualize these variations. Future studies utilizing larger, sex-balanced mammalian cohorts with unified epigenetic markers and detailed endocrine profiling are needed to formally validate potential sex-specific epigenetic patterns.

Despite these limitations, the study provides a focused comparative framework for evaluating whether human OBS-associated methylation signals show similar age-related behavior across mammals. Rather than indicating universal conservation, the results point to partial preservation alongside substantial species-dependent divergence. This framework may be useful for prioritizing candidate loci for future mechanistic and translational studies of redox-related epigenetic aging.

## 4. Materials and Methods

### 4.1. Study Population

This study utilized data from the ASAS cohorts of the KoGES, a longitudinal prospective study initiated in 2001 [15]. The baseline survey included detailed food frequency questionnaires (FFQ) and lifestyle assessments (Table 1).

#### 4.1.1. OBS Calculation

All statistical analyses were performed in the R (version 4.5.1) environment. The OBS was calculated by summing the scores of 13 pro-oxidant and antioxidant components, following established methodologies [3,4]. Higher scores indicate a predominance of antioxidant exposure. Genomic DNA was extracted from peripheral blood leukocytes, and DNA methylation was profiled using the Illumina Infinium HumanMethylation450 BeadChip [22]. Raw IDAT files were processed using the *minfi* R package (version 1.48.0) [23]. To correct for background signal and dye bias, the data were normalized with the preprocessNoob() function [24]. Rigorous quality-control filters were applied: probes with a detection *p*-value ≥ 0.01 or a bead count < 3 were excluded from downstream analyses. Potential batch effects—particularly those arising from chip and plate configurations—were examined, and intensity disparities were reduced through the same Noob normalization step. After this stringent filtering, the retained CpG sites numbered 485,512 for the ASAS baseline cohort (450K array, *n* = 400). Beta values were normalized using the Beta Mixture Quantile dilation (BMIQ) method [25].

#### 4.1.2. Covariate-Adjusted EWAS

To robustly identify OBS-associated CpGs while controlling for potential confounders, Beta values were converted to M-values to satisfy the assumptions of homoscedasticity for linear modeling. Blood cell-type proportions (CD8T, CD4T, NK, B cell, monocytes, and granulocytes) were estimated using the FlowSorted.Blood.450k packages (version 1.51.0). A covariate-adjusted epigenome-wide association study (EWAS) was conducted using the limma package (version 3.60.0), adjusting for OBS and the estimated cell-type proportions. Statistical significance was defined using the Benjamini–Hochberg False Discovery Rate (FDR < 0.05).

### 4.2. Cross-Species Data Processing

To investigate evolutionary conservation, we utilized the Mammalian Methylation Consortium dataset (GSE223748) [16,17]. This custom array platform targets approximately 37,000 CpG sites highly conserved across mammalian species, allowing for direct orthologous comparison. We mapped the 26 OBS-associated human CpG sites to this dataset by exactly matching their unique Illumina ‘cg’ identifiers (cg#s). We deliberately did not restrict our initial human EWAS to these 37,000 conserved markers in order to allow for an unbiased discovery of human redox-related epigenetic signatures [26], which might include human-specific adaptations. Instead, we cross-referenced our unbiased human findings with the mammalian dataset secondarily to determine whether these prioritized human markers exhibit preserved age-related behaviors across other mammals. The analysis included nine representative species covering major mammalian clades: Primates (*Homo sapiens*, *Macaca mulatta*), Rodentia (*Mus musculus*, *Rattus norvegicus*), Carnivora (*Canis lupus familiaris*, *Felis catus*), Perissodactyla (*Equus caballus*), and Artiodactyla (*Capreolus capreolus*, *Ovis aries*). Since direct OBS data is unavailable for non-human species, “chronological age” was used as a proxy variable for cumulative oxidative stress, based on the fundamental biological premise that oxidative damage accumulates linearly or exponentially with age [1,2].

### 4.3. Statistical Analysis and Visualization

All statistical analyses were performed in the R (version 4.5.1) environment [27]. The R-based source code for this process is available on GitHub (https://github.com/vitastarz-jpg/gimlab) (accessed on 22 September 2026).

#### 4.3.1. Correlation and Clustering Analysis

To assess the relationship between DNA methylation levels and OBS, as well as age-related correlations across species, we calculated both Pearson (rp) and Spearman (rs) correlation coefficients using the base R function cor() with the argument use = “pairwise.complete.obs”. Hierarchical clustering of these correlation profiles was performed and visualized using the pheatmap() function from the pheatmap package (version 1.0.13) [28]. To strictly identify robust OBS-associated epigenetic markers and minimize false discoveries, we applied a multi-step analytical pipeline. First, CpG sites were initially screened if they simultaneously exhibited an absolute correlation coefficient greater than 0.23 for both methods (|rp| > 0.23 and |rs| > 0.23), with corresponding nominal *p*-values < 0.003. Second, to ensure that the associations were independent of blood cell composition, the pre-filtered CpGs were subjected to a covariate-adjusted multiple linear regression model. By adjusting for estimated cell-type proportions (CD8T, CD4T, NK, B cells, and monocytes), we effectively removed potential confounding artifacts. Only CpG sites that satisfied the False Discovery Rate (FDR < 0.05) threshold in this linear model were finally retained. To systematically prioritize these markers for cross-species evolutionary analysis, we computed a custom “Hybrid Pi-score (HyPi)” to integrate both the correlation effect sizes and statistical significance into a single metric. The HyPi score was defined as follows:

*HyPi* = (*r_p_* × −log_10_ (*P_p_*)) + (*r_s_* × −log_10_ (*P_s_*))

Sites were then ranked by this HyPi metric, and their orthologous conservation was evaluated across the Mammalian Methylation Consortium dataset.

#### 4.3.2. Functional Pathway Analysis by QIAGEN IPA

To elucidate the functional relevance of the identified 26 phylogenetically conserved CpG sites and bridge these epigenetic markers to systemic oxidative biology, in silico pathway analysis was conducted using QIAGEN Ingenuity Pathway Analysis (IPA; QIAGEN Inc., Redwood City, CA, USA) [29].

A Core Analysis was performed using the Ingenuity Knowledge Base as the reference set, restricted to human, mouse, and rat species to optimally reflect the mammalian evolutionary context. We systematically queried ‘Canonical Pathways’, and ‘Diseases and Bio Functions’, to map these localized upstream sensors to macro-level cellular effector networks.

#### 4.3.3. Evolutionary Trend Visualization

The linearity between Pearson and Spearman coefficients was assessed using scatter plots generated with the ggplot() function from the ggplot2 package (version 4.0.3) [30]. To visualize the “Phylo-Correlation Decay,” we aggregated the absolute correlation values by species and plotted the decay trends using stat_summary(fun = mean, geom = “line”). Species-specific distributions of correlation coefficients were visualized using boxplots combined with jittered points via geom_boxplot() and geom_jitter() to display individual CpG variability. The consensus versus divergence of methylation patterns relative to humans was analyzed by plotting species-specific correlations against human correlations, fitted with linear regression models using geom_smooth(method = “lm”).

#### 4.3.4. Multivariate Analysis and Epigenetic Phylogeny

To explore the epigenetic landscape, we performed Principal Component Analysis (PCA) on the transposed correlation matrices using the prcomp() function with centering and scaling [31]. Additionally, Multidimensional Scaling (MDS) was conducted using the cmdscale() function to project epigenetic distances into two-dimensional space [32].

For phylogenetic reconstruction, we first calculated the epigenetic distance between species based on their methylation correlation profiles using the dist() function with method = “euclidean”. Subsequently, unrooted phylogenetic trees were constructed using the Neighbor-Joining (NJ) method [33] via the nj() function from the ape package (version 5.8) [34].

To structurally compare the epigenetic trees of males and females, we converted the hierarchical clusters into dendrogram objects using as.dendrogram() and hclust(). The alignment and entanglement of these sex-specific trees were visualized using the tanglegram() function from the dendextend package (version 1.19.1) [35].

#### 4.3.5. Phylogenetic Sensitivity Analysis

To validate the topological stability of the reduced-feature Neighbor-Joining (NJ) trees, 1000-iteration bootstrap resampling was performed utilizing the ape package, with node support values visualized on the respective branches. Additionally, a null-model sensitivity analysis was conducted to ensure the phylogenetic patterns were not driven by random background noise. Size-matched random CpG sets were sampled 1000 times from the conserved mammalian array space, and the Robinson–Foulds (RF) topological distances between the random trees and the true OBS-tree were calculated using the dist.topo() function.

## 5. Conclusions

In conclusion, we identified 26 CpG sites associated with OBS in the KoGES cohort and showed that these human-prioritized loci display heterogeneous age-related correlation patterns across mammalian species. The overall pattern was more similar between humans and rhesus macaques than between humans and several non-primate species, consistent with partial evolutionary conservation of a subset of human OBS-associated methylation signals. These findings support the use of comparative epigenetic analysis as a framework for distinguishing CpG sites that appear relatively conserved from those that are more species-dependent, while also underscoring the need for cautious interpretation when human phenotype-based markers are evaluated across species using age as a proxy.

## Figures and Tables

**Table 1:** Participant information.

Variable	Male (*n* = 169)	Female (*n* = 173)
Age (Year)	51.1 ± 7.8	54.1 ± 8.5
BMI (kg/m^2^)	24.2 ± 3.3	25.2 ± 3.6
Exercise (MET *-hours/day)	74.3 ± 39.6	73.1 ± 40.7
Drinking (g/day)	19.4 ± 27.9	1.6 ± 6.7
Smoking (Non-smoker/Former smoker/Current smoker)	35/49/85	168/1/4
Fiber (g)	6.9 ± 2.8	7.4 ± 3.6
Carotene (µg)	2735.3 ± 1983.3	2537.7 ± 2002.4
Vitamin B2 (mg)	1 ± 0.4	0.9 ± 0.5
Niacin (mg)	16.8 ± 5.2	14.5 ± 6.6
Vitamin B6 (mg)	1.8 ± 0.6	1.8 ± 0.9
Folate (µg)	244.6 ± 104.3	241.4 ± 129.3
Vitamin C (mg)	118.1 ± 72.2	139.7 ± 106.7
Vitamin E (mg)	9.8 ± 4.6	9.4 ± 8.2
Calcium (mg)	475.2 ± 216	457.7 ± 267.3
Zinc (mg)	9.3 ± 3.1	8.2 ± 4.2
Fat (g)	37.5 ± 17.1	27.6 ± 20.7
Iron (mg)	11.2 ± 4.1	10.7 ± 5.3

* MET: Metabolic equivalent of task.

**Table 2:** Annotation and correlation characteristics of the 26 OBS-associated CpG sites identified in the KoGES cohort.

ID	Chr	Location *	Gene Symbol	Genomic Loc.	Cellular Loc.	Type	CpG Island	SEX	Coef **	PV ***	FDR ***	HyPi **
cg00727310	2	227,662,704	*IRS1*	1stExon			Island	M	0.011	0.00137	0.00218	1.419
cg02132051	14	73,020,007	*RGS6*	Body				M	0.012	0.00021	0.00112	2.497
cg02499843	7	132,261,287	*PLXNA4*	1stExon			Island	M	0.011	0.00308	0.00308	1.421
cg02650266	4	147,558,239					Island	F	−0.02	0.001	0.00218	−1.403
cg03486383	16	87,865,062	*SLC7A5*	3’UTR				M	0.01	0.00192	0.00222	1.428
cg03846926	10	21,807,211	*C10orf140*	5’UTR			S_Shore	F	−0.008	0.00235	0.00243	−1.429
cg04218899	1	183,155,366	*LAMC2*	1stExon				F	−0.02	0.00014	0.00112	−1.73
cg04388989	19	30,715,606					Island	M	0.017	0.00171	0.00218	1.446
cg05486213	11	30,607,950	*MPPED2*	TSS200			Island	M	0.017	0.00013	0.00112	1.647
cg05844937	6	1,614,141					Island	M	0.009	0.00211	0.00227	1.218
cg06458239	19	58,038,573	*ZNF549*	TSS200			Island	F	−0.014	0.00022	0.00112	−1.362
cg06635150	5	171,094,937						F	0.008	0.00162	0.00218	1.622
cg07604651	7	121,944,755	*FEZF1*	TSS200			N_Shore	M	0.012	0.00168	0.00218	1.434
cg10944833	6	17,702,460	*NUP153*	Body			N_Shelf	F	−0.012	0.00065	0.00207	−1.441
cg11476211	2	45,879,284	*PRKCE*	1stExon			Island	F	−0.011	0.00076	0.00207	−1.819
cg11932564	22	42,322,146	*TNFRSF13C*	Body			Island	M	0.01	0.0016	0.00218	1.513
cg16393928	1	3,135,836	*PRDM16*	Body				M	0.01	0.00109	0.00218	1.592
cg16434657	3	137,490,228					Island	F	−0.011	0.00159	0.00218	−1.394
cg16802592	15	37,179,603	*LOC145845*	TSS1500			Island	M	0.011	0.00139	0.00218	1.749
cg18397523	7	27,282,351	*EVX1*	1stExon			Island	F	−0.009	0.00187	0.00222	−1.27
cg18930863	11	117,389,197	*DSCAML1*	Body			Island	M	0.008	0.00175	0.00218	1.334
cg20014398	11	31,825,792	*PAX6*	Body			Island	F	−0.016	0.00074	0.00207	−1.397
cg20550118	15	78,633,662	*CRABP1*	Body			Island	F	−0.013	0.00017	0.00112	−2.078
cg25925945	2	183,902,764	*NCKAP1*	1stExon			Island	F	−0.012	0.00034	0.00126	−1.807
cg26372517	1	36,039,159	*TFAP2E*	5’UTR			Island	F	−0.02	0.00171	0.00218	−1.636
cg26880891	15	72,767,139	*ARIH1*	1stExon			Island	F	−0.008	0.00172	0.00218	−1.539

* Genome assembly version: hg19, ** Rounded to three decimal places, *** Rounded to five decimal places.

**Table 3:** Predicted functional associations of the annotated OBS-associated CpG sites based on Ingenuity Pathway Analysis (IPA). IPA was used as a hypothesis-generating tool to map host genes to canonical pathways and biological functions.

Categories	Annotation	*p*-Value	Z-Score	Genes	No. of Genes
Gene Expression	Transcription of gene	0.000206	0.219	*EVX1*, *FEZF1*, *NUP153*, *PAX6*, *PRDM16*, *PRKCE*	6
Cell Death and Survival, Organismal Injury and Abnormalities	Apoptosis of lung cancer cell lines	0.00101	0.32	*IRS1*, *LAMC2*, *PLXNA4*, *PRKCE*	4
Cellular Development, Cellular Growth and Proliferation	Cell proliferation of tumor cell lines	0.0034	0.779	*ARIH1*, *FEZF1*, *IRS1*, *LAMC2*, *PAX6*, *PLXNA4*, *PRDM16*, *PRKCE*, *SLC7A5*, *TNFRSF13C*	10
Cellular Function and Maintenance, Protein Synthesis	Metabolism of protein	0.00593	0.178	*ARIH1*, *FEZF1*, *IRS1*, *LAMC2*, *NCKAP1*, *PAX6*, *PLXNA4*, *PRDM16*, *PRKCE*, *RGS6*, *SLC7A5*, *TNFRSF13C*	12
Cellular Development, Cellular Growth and Proliferation	Proliferation of lung cancer cell lines	0.0085	−0.647	*IRS1*, *LAMC2*, *PRKCE*, *SLC7A5*	4
Gene Expression	Transcription of RNA	0.0092	−0.16	*EVX1*, *FEZF1*, *NUP153*, *PAX6*, *PRDM16*, *PRKCE*	6
Cell Death and Survival, Organismal Injury and Abnormalities	Apoptosis of carcinoma cell lines	0.0136	0.475	*IRS1*, *LAMC2*, *PLXNA4*, *PRKCE*	4
Cellular Function and Maintenance, Protein Degradation, Protein Synthesis	Catabolism of protein	0.0207	−0.14	*ARIH1*, *IRS1*, *LAMC2*, *NCKAP1*, *PRKCE*, *TNFRSF13C*	6
Post-Translational Modification	Ubiquitination of protein	0.0267	0.243	*ARIH1*, *IRS1*, *LAMC2*, *SLC7A5*	4
Cellular Development, Cellular Growth and Proliferation	Cell proliferation of carcinoma cell lines	0.0429	0.366	*FEZF1*, *IRS1*, *LAMC2*, *PRKCE*, *SLC7A5*	5

## Data Availability

The R source codes used for the analysis are available on GitHub (https://github.com/vitastarz-jpg/gimlab (accessed on 22 September 2026), Loading_TCGA_RNAseq_Met_Common_eQTL.R and OBS_Evolution_IJMS_2026.R). The GSE223748 dataset can be download from the NCBI GEO (https://www.ncbi.nlm.nih.gov/geo/query/acc.cgi?acc=GSE223748, accessed on 22 September 2026). The KoGES dataset can be obtained through a process established by the Ministry of Health and Welfare of the Republic of Korea, following approval from the institution’s Institutional Review Board (IRB) (https://www.nih.go.kr/ko/main/contents.do?menuNo=300566, accessed on 22 September 2026).

## Supplementary Materials

The following supporting information can be downloaded at: https://www.mdpi.com/article/10.3390/ijms27198772/s1.

## Abbreviations

The following abbreviations are used in this manuscript:

ASAS	Ansan-Ansung (cohort)
CpG	Cytosine-phosphate-Guanine
EWAS	Epigenome-Wide Association Study
HyPi	Hybrid Pi-score
IPA	Ingenuity Pathway Analysis
KoGES	Korean Genome and Epidemiology Study
MDS	Multidimensional Scaling
OBS	Oxidative Balance Score
PCA	Principal Component Analysis
ROS	Reactive Oxygen Species
