# Supplementary material for: Evolutionary Epigenetic Analysis of Oxidative Balance Score-Associated DNA Methylation Sites Across Mammals
Source: Int J Mol Sci. 2026 Sep 30;27(19):8772. doi: 10.3390/ijms27198772 (PMC13664758; doi:10.3390/ijms27198772)
Supplement: Supplementary file 1 [file ijms-27-08772-s001.zip › ijms-4447199-supplementary.pdf]

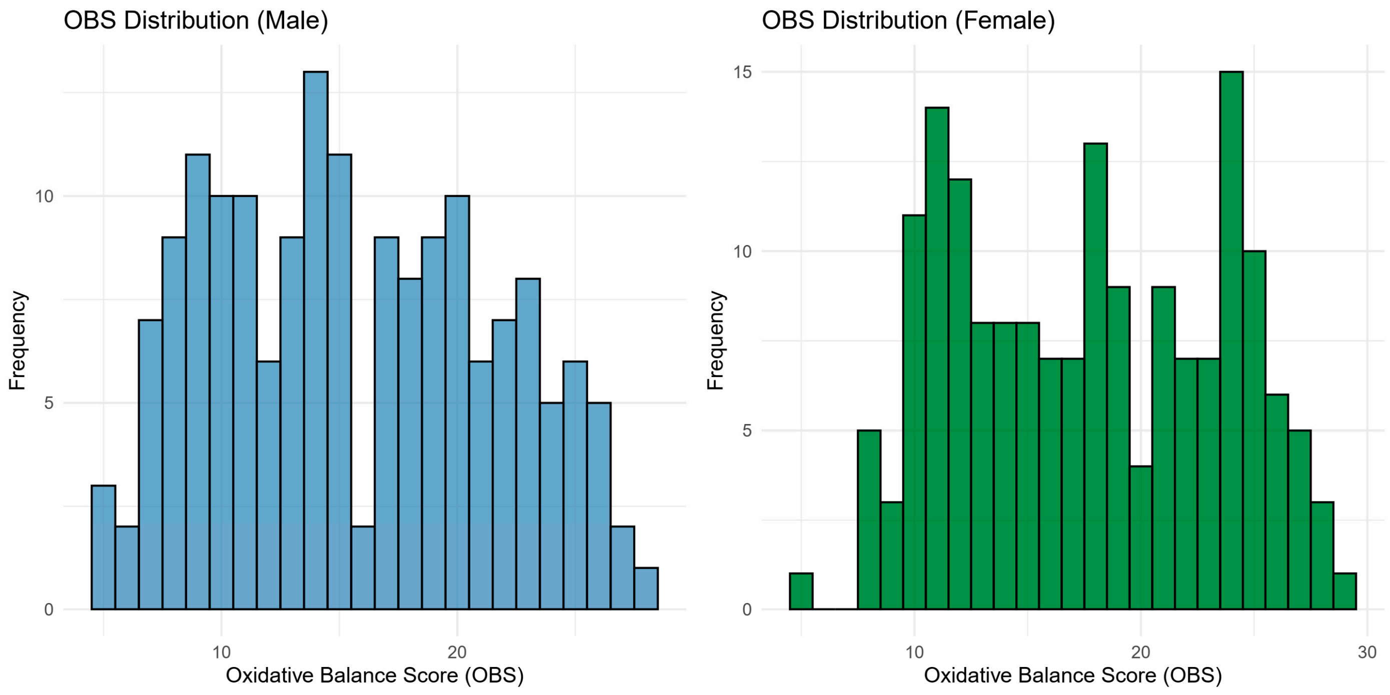

Figure S1. Histograms showing the frequency distribution of the Oxidative Balance Score (OBS) in the male and female KoGES ASAS cohorts.

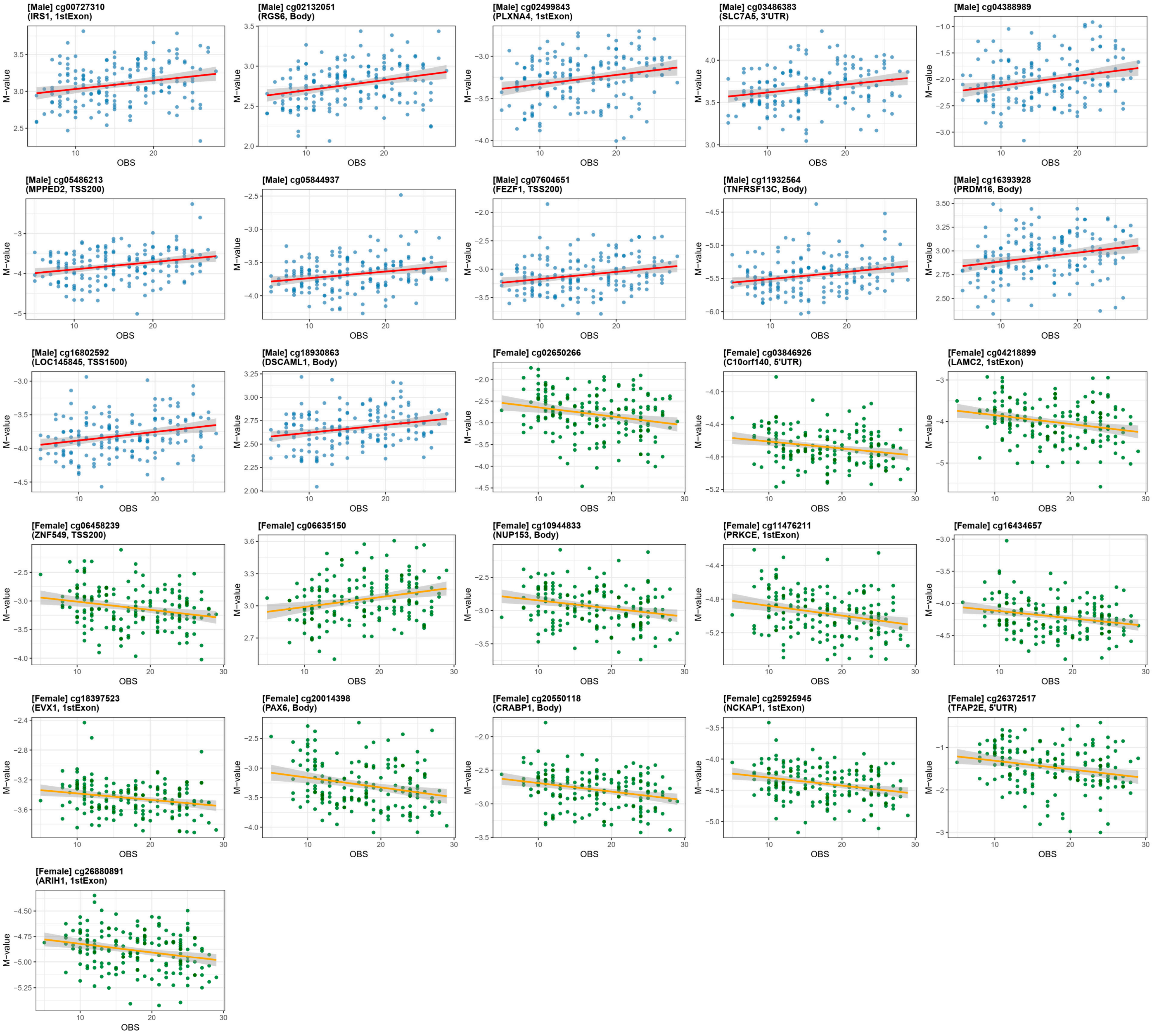

Figure S2. Scatter plots of DNA methylation M-values versus Oxidative Balance Score (OBS) for the 26 identified CpG sites in the KoGES ASAS cohort. Red lines (males) and orange lines (females) indicate the linear regression trends.

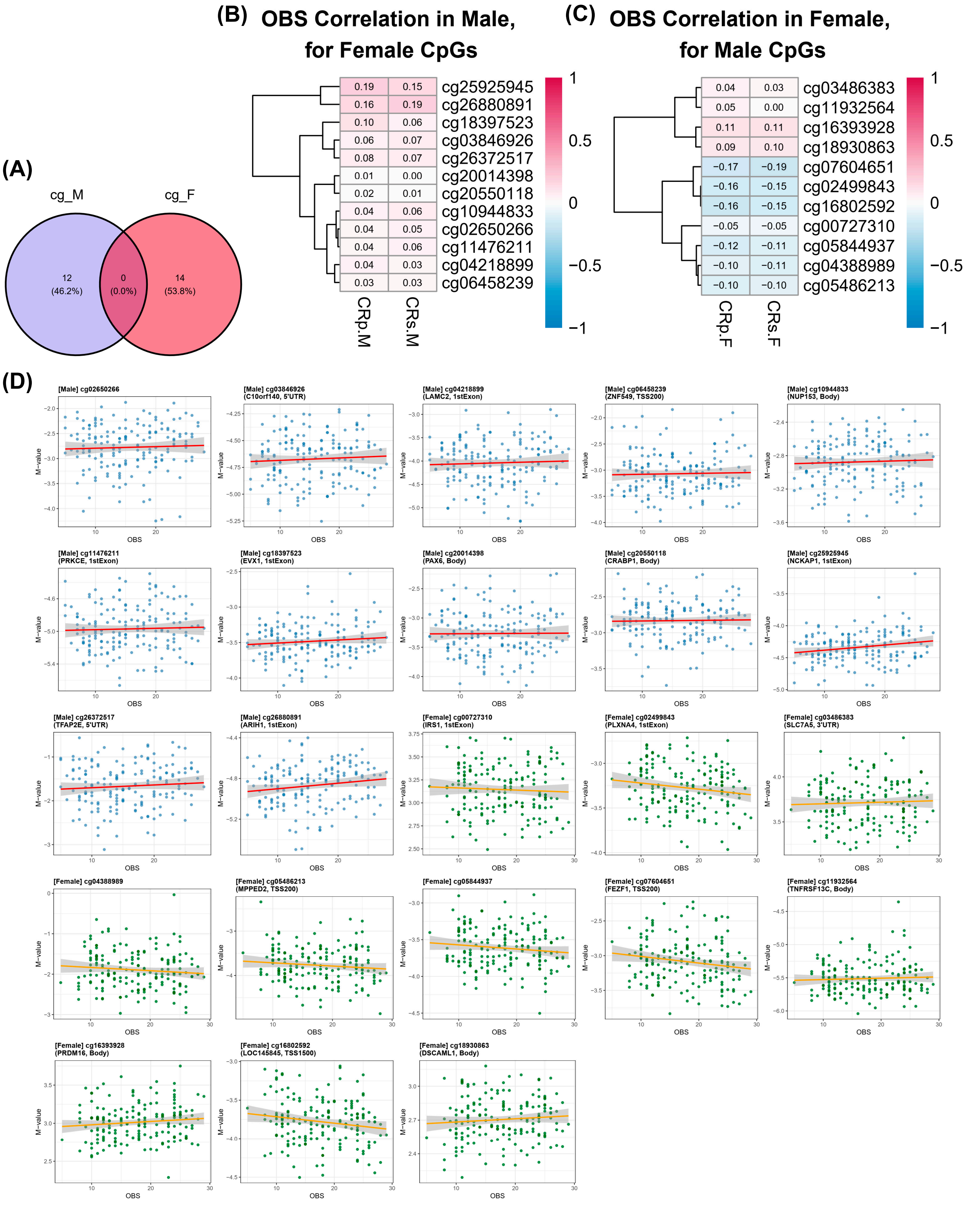

Figure S3. Sex-specific association patterns of OBS-related CpGs. (A) Venn diagram illustrating the absence of overlap (0.0%) between the significantly associated CpGs identified in males (n=12) and females (n=14). (B) Heatmap displaying the Pearson (CRp.M) and Spearman (CRs.M) correlation coefficients of the 12 female-specific CpGs when analyzed within the male cohort. (C) Heatmap displaying the Pearson (CRp.F) and Spearman (CRs.F) correlation coefficients of the 11 male-specific CpGs when analyzed within the female cohort. The color gradient indicates the strength and direction of the correlation, with red representing positive and blue representing negative correlations. (D) Scatter plots illustrating DNA methylation M-values versus Oxidative Balance Score (OBS) for the 23 sex-specific CpG sites in the KoGES ASAS cohort, showing the results of cross-sex analysis. Red lines (males) and orange lines (females) indicate the linear regression trends.

Table S1. Expression quantitative trait methylation (eQTM) analysis of OBS-associated CpGs across 31 TCGA cancer cohorts. The table summarizes the Pearson and Spearman correlation coefficients evaluating the relationship between DNA methylation levels at specific OBS-associated CpG sites and the mRNA expression of their corresponding host genes. Data were derived from matched TCGA samples. Significant inverse correlations (negative R values) indicate potential epigenetic silencing of the host gene driven by regional hypermethylation. The Hybrid Pi-score (HyPi) integrates both the correlation effect sizes and nominal P-values to quantify the overall strength and directionality of the association.

Cancer	ID	Gene	Pearson_R	Pearson_P	Spearman_R	Spearman_P	HyPi
ACC	cg00727310	IRS1	-0.297	0.008	-0.288	0.01	-1.198
ACC	cg02499843	PLXNA4	-0.104	0.36	-0.089	0.435	-0.079
ACC	cg03486383	SLC7A5	0.073	0.52	-0.083	0.468	-0.006
ACC	cg04218899	LAMC2	-0.279	0.013	-0.471	0	-2.844
ACC	cg05486213	MPPED2	-0.236	0.037	-0.095	0.403	-0.376
ACC	cg06458239	ZNF549	-0.097	0.393	-0.329	0.003	-0.865
ACC	cg07604651	FEZF1	0.18	0.113	0.118	0.302	0.232
ACC	cg10944833	NUP153	-0.056	0.627	-0.083	0.466	-0.039
ACC	cg11476211	PRKCE	0.044	0.701	-0.022	0.847	0.005
ACC	cg11932564	TNFRSF13C	-0.138	0.226	-0.112	0.324	-0.144
ACC	cg16393928	PRDM16	0.114	0.317	0.197	0.082	0.271
ACC	cg18397523	EVX1	-0.323	0.004	-0.402	0	-2.243
ACC	cg18930863	DSCAML1	0.001	0.991	-0.129	0.256	-0.076
ACC	cg20014398	PAX6	0.296	0.008	0.341	0.002	1.529
ACC	cg20550118	CRABP1	0.185	0.102	0.072	0.529	0.203
ACC	cg25925945	NCKAP1	-0.015	0.898	-0.026	0.819	-0.003
ACC	cg26372517	TFAP2E	0.261	0.02	0.171	0.132	0.594
BLCA	cg00727310	IRS1	-0.467	0	-0.462	0	-20.671
BLCA	cg02499843	PLXNA4	-0.012	0.812	0.1	0.045	0.133
BLCA	cg03486383	SLC7A5	0.111	0.026	0.019	0.699	0.178
BLCA	cg04218899	LAMC2	-0.068	0.171	0.061	0.218	-0.012
BLCA	cg05486213	MPPED2	-0.176	0	-0.285	0	-2.945
BLCA	cg06458239	ZNF549	-0.523	0	-0.454	0	-24.725
BLCA	cg07604651	FEZF1	-0.157	0.002	-0.23	0	-1.708
BLCA	cg10944833	NUP153	-0.022	0.653	-0.005	0.922	-0.004
BLCA	cg11476211	PRKCE	-0.132	0.008	-0.024	0.632	-0.282
BLCA	cg11932564	TNFRSF13C	-0.12	0.016	-0.142	0.004	-0.55
BLCA	cg16393928	PRDM16	0.217	0	0.376	0	6.427
BLCA	cg18397523	EVX1	-0.217	0	-0.274	0	-3.187
BLCA	cg18930863	DSCAML1	0.145	0.003	0.233	0	1.666
BLCA	cg20014398	PAX6	0.265	0	0.211	0	2.893
BLCA	cg20550118	CRABP1	0.246	0	0.15	0.002	1.93
BLCA	cg25925945	NCKAP1	0.027	0.59	0.084	0.093	0.093
BLCA	cg26372517	TFAP2E	-0.042	0.4	-0.028	0.575	-0.023
BRCA	cg00727310	IRS1	-0.408	0	-0.366	0	-21.535
BRCA	cg02499843	PLXNA4	-0.008	0.825	0.026	0.485	0.007
BRCA	cg03486383	SLC7A5	-0.071	0.051	-0.143	0	-0.669
BRCA	cg04218899	LAMC2	-0.276	0	-0.193	0	-5.171
BRCA	cg05486213	MPPED2	-0.116	0.001	-0.134	0	-0.817
BRCA	cg06458239	ZNF549	-0.473	0	-0.405	0	-32.246
BRCA	cg10944833	NUP153	-0.033	0.369	-0.022	0.543	-0.02
BRCA	cg11476211	PRKCE	-0.244	0	-0.337	0	-9.649
BRCA	cg11932564	TNFRSF13C	-0.144	0	-0.198	0	-2.038
BRCA	cg16393928	PRDM16	0.137	0	0.293	0	5.076
BRCA	cg18397523	EVX1	0.14	0	0.221	0	2.536
BRCA	cg18930863	DSCAML1	0.138	0	0.209	0	2.235
BRCA	cg20014398	PAX6	0.14	0	0.109	0.003	0.827
BRCA	cg20550118	CRABP1	-0.159	0	-0.117	0.001	-1.113
BRCA	cg25925945	NCKAP1	0.04	0.271	0.065	0.077	0.095
BRCA	cg26372517	TFAP2E	-0.004	0.915	0.071	0.052	0.091
CESC	cg00727310	IRS1	-0.119	0.041	-0.088	0.134	-0.242
CESC	cg02499843	PLXNA4	-0.174	0.003	-0.147	0.012	-0.727
CESC	cg03486383	SLC7A5	-0.011	0.854	-0.029	0.624	-0.007
CESC	cg04218899	LAMC2	-0.092	0.117	0	0.996	-0.085
CESC	cg05486213	MPPED2	-0.24	0	-0.383	0	-5.272
CESC	cg06458239	ZNF549	-0.838	0	-0.793	0	-116.29
CESC	cg10944833	NUP153	0.035	0.556	0.033	0.576	0.017
CESC	cg11476211	PRKCE	-0.027	0.645	-0.105	0.073	-0.124

CESC	cg11932564	TNFRSF13C	0.079	0.178	-0.074	0.208	0.009
CESC	cg16393928	PRDM16	-0.083	0.154	0.084	0.151	0.002
CESC	cg18397523	EVX1	-0.105	0.071	-0.071	0.223	-0.168
CESC	cg18930863	DSCAML1	0.038	0.515	0.009	0.873	0.012
CESC	cg20014398	PAX6	0.316	0	0.334	0	5.165
CESC	cg20550118	CRABP1	-0.093	0.113	-0.097	0.098	-0.185
CESC	cg25925945	NCKAP1	-0.008	0.897	0.027	0.644	0.005
CESC	cg26372517	TFAP2E	-0.153	0.009	-0.08	0.17	-0.378
CHOL	cg00727310	IRS1	-0.299	0.076	-0.321	0.056	-0.737
CHOL	cg02499843	PLXNA4	-0.139	0.419	0.022	0.898	-0.052
CHOL	cg03486383	SLC7A5	-0.229	0.18	-0.161	0.349	-0.244
CHOL	cg04218899	LAMC2	-0.525	0.001	0.221	0.196	-1.412
CHOL	cg05486213	MPPED2	-0.153	0.374	-0.002	0.992	-0.065
CHOL	cg06458239	ZNF549	-0.767	0	-0.688	0	-9.367
CHOL	cg07604651	FEZF1	-0.176	0.305	-0.068	0.691	-0.102
CHOL	cg10944833	NUP153	-0.323	0.055	-0.335	0.046	-0.855
CHOL	cg11476211	PRKCE	-0.129	0.454	0.24	0.159	0.147
CHOL	cg11932564	TNFRSF13C	-0.117	0.495	-0.032	0.853	-0.038
CHOL	cg16393928	PRDM16	-0.238	0.162	-0.146	0.397	-0.247
CHOL	cg18397523	EVX1	0.116	0.5	0.337	0.045	0.489
CHOL	cg18930863	DSCAML1	0.192	0.262	0.268	0.114	0.364
CHOL	cg20014398	PAX6	0.222	0.194	0.288	0.089	0.46
CHOL	cg20550118	CRABP1	-0.285	0.092	-0.218	0.202	-0.446
CHOL	cg25925945	NCKAP1	-0.323	0.055	-0.158	0.357	-0.478
CHOL	cg26372517	TFAP2E	0.03	0.863	0.174	0.31	0.09
COAD	cg00727310	IRS1	-0.329	0	-0.345	0	-5.612
COAD	cg02499843	PLXNA4	-0.203	0.001	-0.189	0.001	-1.205
COAD	cg03486383	SLC7A5	-0.076	0.203	-0.091	0.126	-0.134
COAD	cg04218899	LAMC2	0.14	0.018	0.195	0.001	0.837
COAD	cg05486213	MPPED2	-0.248	0	-0.206	0	-1.84
COAD	cg06458239	ZNF549	-0.828	0	-0.818	0	-116.858
DLBC	cg00727310	IRS1	-0.468	0.001	-0.345	0.018	-2.025
DLBC	cg02499843	PLXNA4	-0.257	0.081	-0.272	0.064	-0.605
DLBC	cg03486383	SLC7A5	-0.199	0.18	-0.242	0.101	-0.39
DLBC	cg04218899	LAMC2	-0.357	0.014	-0.308	0.035	-1.114
DLBC	cg05486213	MPPED2	-0.203	0.171	-0.202	0.174	-0.309
DLBC	cg06458239	ZNF549	-0.704	0	-0.639	0	-9.02
DLBC	cg07604651	FEZF1	-0.289	0.049	-0.12	0.421	-0.424
DLBC	cg10944833	NUP153	-0.218	0.141	-0.209	0.158	-0.353
DLBC	cg11476211	PRKCE	-0.446	0.002	-0.296	0.043	-1.641
DLBC	cg11932564	TNFRSF13C	0.292	0.047	0.356	0.014	1.048
DLBC	cg16393928	PRDM16	-0.346	0.017	-0.297	0.042	-1.019
DLBC	cg18397523	EVX1	-0.235	0.112	-0.198	0.182	-0.371
DLBC	cg18930863	DSCAML1	0.059	0.695	0.134	0.368	0.068
DLBC	cg20014398	PAX6	-0.268	0.069	0.003	0.984	-0.311
DLBC	cg20550118	CRABP1	0.195	0.19	0.228	0.124	0.347
DLBC	cg25925945	NCKAP1	-0.223	0.132	-0.172	0.249	-0.299
DLBC	cg26372517	TFAP2E	0.02	0.895	-0.006	0.968	0.001
ESCA	cg00727310	IRS1	-0.377	0	-0.368	0	-4.196
ESCA	cg02499843	PLXNA4	-0.104	0.201	-0.191	0.019	-0.403
ESCA	cg03486383	SLC7A5	-0.112	0.168	-0.171	0.036	-0.334
ESCA	cg04218899	LAMC2	-0.206	0.011	-0.123	0.13	-0.512
ESCA	cg05486213	MPPED2	-0.335	0	-0.297	0	-2.636
ESCA	cg06458239	ZNF549	-0.689	0	-0.758	0	-37.071
ESCA	cg10944833	NUP153	-0.019	0.82	0.01	0.904	-0.001
ESCA	cg11476211	PRKCE	-0.227	0.005	-0.117	0.15	-0.622
ESCA	cg11932564	TNFRSF13C	0.041	0.617	0.016	0.848	0.01
ESCA	cg16393928	PRDM16	-0.244	0.002	-0.131	0.107	-0.762
ESCA	cg18397523	EVX1	-0.021	0.8	0.197	0.015	0.357
ESCA	cg18930863	DSCAML1	0.139	0.087	0.233	0.004	0.709
ESCA	cg20014398	PAX6	0.288	0	0.285	0	1.981
ESCA	cg20550118	CRABP1	-0.023	0.774	-0.236	0.003	-0.588
ESCA	cg25925945	NCKAP1	-0.036	0.663	-0.043	0.603	-0.016
ESCA	cg26372517	TFAP2E	-0.152	0.062	-0.044	0.589	-0.193
GBM	cg00727310	IRS1	0.044	0.774	-0.086	0.576	-0.016
GBM	cg02499843	PLXNA4	-0.161	0.29	-0.172	0.259	-0.188

GBM	cg03486383	SLC7A5	-0.018	0.908	-0.026	0.863	-0.002
GBM	cg04218899	LAMC2	-0.095	0.533	-0.164	0.283	-0.116
GBM	cg05486213	MPPED2	-0.264	0.079	-0.221	0.144	-0.478
GBM	cg06458239	ZNF549	-0.37	0.012	-0.446	0.002	-1.898
GBM	cg07604651	FEZF1	-0.169	0.268	-0.148	0.331	-0.167
GBM	cg10944833	NUP153	0.362	0.014	0.302	0.044	1.078
GBM	cg11476211	PRKCE	0.022	0.885	-0.125	0.412	-0.047
GBM	cg11932564	TNFRSF13C	0.09	0.558	0.017	0.914	0.023
GBM	cg16393928	PRDM16	-0.229	0.131	-0.101	0.508	-0.232
GBM	cg18397523	EVX1	0.019	0.902	-0.132	0.386	-0.054
GBM	cg18930863	DSCAML1	-0.017	0.912	0.045	0.768	0.005
GBM	cg20014398	PAX6	-0.034	0.825	0.111	0.47	0.033
GBM	cg20550118	CRABP1	0.132	0.387	0.19	0.21	0.183
GBM	cg25925945	NCKAP1	-0.113	0.462	-0.145	0.343	-0.105
GBM	cg26372517	TFAP2E	0.359	0.015	0.175	0.251	0.757
HNSC	cg00727310	IRS1	-0.417	0	-0.39	0	-16.228
HNSC	cg02499843	PLXNA4	-0.055	0.223	-0.018	0.696	-0.039
HNSC	cg03486383	SLC7A5	-0.087	0.054	-0.052	0.252	-0.14
HNSC	cg04218899	LAMC2	0.064	0.156	0.063	0.161	0.102
HNSC	cg05486213	MPPED2	-0.209	0	-0.381	0	-7.923
HNSC	cg06458239	ZNF549	-0.697	0	-0.714	0	-105.731
HNSC	cg07604651	FEZF1	-0.254	0	-0.51	0	-19.051
HNSC	cg10944833	NUP153	0.022	0.633	0.026	0.571	0.01
HNSC	cg11476211	PRKCE	-0.204	0	-0.264	0	-3.354
HNSC	cg11932564	TNFRSF13C	-0.225	0	-0.299	0	-4.705
HNSC	cg16393928	PRDM16	0.145	0.001	0.169	0	1.064
HNSC	cg18397523	EVX1	-0.101	0.024	0.003	0.945	-0.164
HNSC	cg18930863	DSCAML1	0.132	0.003	0.133	0.003	0.662
HNSC	cg20014398	PAX6	0.354	0	0.399	0	13.224
HNSC	cg20550118	CRABP1	-0.164	0	-0.127	0.005	-0.885
HNSC	cg25925945	NCKAP1	0.062	0.166	0.078	0.084	0.133
HNSC	cg26372517	TFAP2E	-0.21	0	-0.177	0	-1.907
KICH	cg00727310	IRS1	0.034	0.788	-0.122	0.335	-0.055
KICH	cg02499843	PLXNA4	-0.033	0.796	0.043	0.735	0.003
KICH	cg03486383	SLC7A5	0.06	0.638	0.092	0.469	0.042
KICH	cg04218899	LAMC2	-0.037	0.772	0.031	0.806	-0.001
KICH	cg05486213	MPPED2	-0.208	0.099	-0.066	0.606	-0.224
KICH	cg06458239	ZNF549	-0.063	0.62	-0.175	0.168	-0.149
KICH	cg07604651	FEZF1	-0.058	0.651	-0.116	0.363	-0.062
KICH	cg10944833	NUP153	-0.073	0.564	-0.033	0.795	-0.022
KICH	cg11476211	PRKCE	-0.118	0.352	0.014	0.911	-0.053
KICH	cg11932564	TNFRSF13C	0.007	0.953	-0.006	0.96	0
KICH	cg16393928	PRDM16	-0.257	0.04	-0.273	0.029	-0.777
KICH	cg18397523	EVX1	-0.086	0.501	-0.073	0.567	-0.044
KICH	cg18930863	DSCAML1	0.042	0.741	-0.149	0.241	-0.087
KICH	cg20014398	PAX6	0.29	0.02	0.148	0.242	0.585
KICH	cg20550118	CRABP1	-0.077	0.547	-0.03	0.815	-0.023
KICH	cg25925945	NCKAP1	0.094	0.46	0.074	0.559	0.05
KICH	cg26372517	TFAP2E	0.161	0.205	0.219	0.082	0.348
KIRC	cg00727310	IRS1	-0.343	0	-0.223	0	-4.155
KIRC	cg02499843	PLXNA4	-0.009	0.88	0.058	0.302	0.03
KIRC	cg03486383	SLC7A5	0.004	0.947	-0.004	0.939	0
KIRC	cg04218899	LAMC2	-0.334	0	-0.42	0	-8.899
KIRC	cg05486213	MPPED2	-0.108	0.055	-0.064	0.262	-0.174
KIRC	cg06458239	ZNF549	-0.396	0	-0.395	0	-9.859
KIRC	cg07604651	FEZF1	-0.023	0.688	0.027	0.634	0.002
KIRC	cg10944833	NUP153	0.121	0.031	0.159	0.005	0.555
KIRC	cg11476211	PRKCE	-0.025	0.655	-0.012	0.828	-0.006
KIRC	cg11932564	TNFRSF13C	0.037	0.518	0.034	0.552	0.019
KIRC	cg16393928	PRDM16	-0.129	0.023	-0.043	0.446	-0.227
KIRC	cg18397523	EVX1	0.233	0	0.273	0	2.706
KIRC	cg18930863	DSCAML1	0.098	0.084	0.014	0.805	0.107
KIRC	cg20014398	PAX6	0.136	0.016	0.017	0.764	0.246
KIRC	cg20550118	CRABP1	-0.001	0.989	0.062	0.277	0.034
KIRC	cg25925945	NCKAP1	0.007	0.902	0.053	0.346	0.025
KIRC	cg26372517	TFAP2E	0.183	0.001	0.227	0	1.516

KIRP	cg00727310	IRS1	-0.181	0.003	-0.09	0.142	-0.535
KIRP	cg02499843	PLXNA4	-0.047	0.449	-0.01	0.87	-0.017
KIRP	cg03486383	SLC7A5	0.011	0.864	0.09	0.145	0.076
KIRP	cg04218899	LAMC2	-0.467	0	-0.283	0	-8.655
KIRP	cg05486213	MPPED2	-0.033	0.591	-0.073	0.232	-0.054
KIRP	cg06458239	ZNF549	-0.466	0	-0.444	0	-13.085
KIRP	cg07604651	FEZF1	0.008	0.902	0.057	0.356	0.026
KIRP	cg10944833	NUP153	0.228	0	0.175	0.004	1.275
KIRP	cg11476211	PRKCE	0.075	0.22	0.072	0.24	0.094
KIRP	cg11932564	TNFRSF13C	0.07	0.252	-0.034	0.578	0.034
KIRP	cg16393928	PRDM16	0.042	0.495	0.335	0	2.58
KIRP	cg18397523	EVX1	0.207	0.001	0.18	0.003	1.105
KIRP	cg18930863	DSCAML1	0.139	0.023	0.109	0.076	0.351
KIRP	cg20014398	PAX6	0.261	0	0.195	0.001	1.815
KIRP	cg20550118	CRABP1	-0.05	0.412	0.084	0.172	0.045
KIRP	cg25925945	NCKAP1	0.137	0.025	0.155	0.011	0.523
KIRP	cg26372517	TFAP2E	0.015	0.801	-0.029	0.638	-0.004
LAML	cg00727310	IRS1	0.238	0.023	0.122	0.249	0.463
LAML	cg02499843	PLXNA4	-0.219	0.037	-0.315	0.002	-1.141
LAML	cg03486383	SLC7A5	-0.045	0.671	-0.035	0.742	-0.012
LAML	cg04218899	LAMC2	-0.072	0.498	-0.124	0.24	-0.099
LAML	cg05486213	MPPED2	-0.088	0.407	0.048	0.649	-0.025
LAML	cg06458239	ZNF549	-0.04	0.705	0.004	0.968	-0.006
LAML	cg07604651	FEZF1	-0.068	0.521	-0.028	0.792	-0.022
LAML	cg10944833	NUP153	0.065	0.541	0.021	0.846	0.019
LAML	cg11476211	PRKCE	-0.316	0.002	-0.401	0	-2.47
LAML	cg11932564	TNFRSF13C	-0.018	0.863	0.063	0.552	0.015
LAML	cg16393928	PRDM16	0.057	0.594	0.002	0.988	0.013
LAML	cg18397523	EVX1	-0.037	0.727	-0.047	0.656	-0.014
LAML	cg18930863	DSCAML1	-0.016	0.878	-0.029	0.784	-0.004
LAML	cg20014398	PAX6	0.236	0.024	0.112	0.292	0.443
LAML	cg20550118	CRABP1	0.394	0	0.269	0.01	2.098
LAML	cg25925945	NCKAP1	-0.068	0.521	-0.174	0.099	-0.193
LAML	cg26372517	TFAP2E	0.367	0	0.401	0	2.906
LGG	cg00727310	IRS1	-0.056	0.216	-0.016	0.717	-0.039
LGG	cg02499843	PLXNA4	0.003	0.953	0.02	0.659	0.004
LGG	cg03486383	SLC7A5	-0.101	0.024	-0.084	0.061	-0.267
LGG	cg04218899	LAMC2	-0.042	0.353	-0.067	0.133	-0.078
LGG	cg05486213	MPPED2	0.011	0.802	-0.002	0.957	0.001
LGG	cg06458239	ZNF549	-0.397	0	-0.55	0	-29.672
LGG	cg07604651	FEZF1	-0.17	0	-0.355	0	-6.129
LGG	cg10944833	NUP153	0.147	0.001	0.202	0	1.489
LGG	cg11476211	PRKCE	0.005	0.917	0.011	0.815	0.001
LGG	cg11932564	TNFRSF13C	0.141	0.002	0.155	0.001	0.9
LGG	cg16393928	PRDM16	0.033	0.465	0.031	0.484	0.021
LGG	cg18397523	EVX1	-0.134	0.003	-0.152	0.001	-0.822
LGG	cg18930863	DSCAML1	-0.052	0.251	-0.054	0.227	-0.066
LGG	cg20014398	PAX6	-0.165	0	-0.127	0.005	-0.904
LGG	cg20550118	CRABP1	-0.125	0.005	-0.055	0.218	-0.322
LGG	cg25925945	NCKAP1	0.067	0.137	0.104	0.02	0.234
LGG	cg26372517	TFAP2E	0.352	0	0.276	0	7.945
LIHC	cg00727310	IRS1	-0.271	0	-0.323	0	-4.955
LIHC	cg02499843	PLXNA4	-0.05	0.342	0.038	0.47	-0.011
LIHC	cg03486383	SLC7A5	-0.029	0.585	-0.062	0.236	-0.046
LIHC	cg04218899	LAMC2	-0.404	0	-0.421	0	-12.987
LIHC	cg05486213	MPPED2	-0.087	0.098	-0.062	0.236	-0.126
LIHC	cg06458239	ZNF549	-0.652	0	-0.711	0	-69.592
LIHC	cg07604651	FEZF1	0.169	0.001	-0.059	0.264	0.457
LIHC	cg10944833	NUP153	-0.068	0.195	0.001	0.982	-0.048
LIHC	cg11476211	PRKCE	-0.103	0.048	-0.107	0.04	-0.286
LIHC	cg11932564	TNFRSF13C	-0.087	0.097	-0.038	0.464	-0.101
LIHC	cg16393928	PRDM16	0.119	0.024	0.278	0	2.178
LIHC	cg18397523	EVX1	0.08	0.129	0.128	0.014	0.308
LIHC	cg18930863	DSCAML1	0.309	0	0.537	0	17.679
LIHC	cg20014398	PAX6	0.281	0	0.296	0	4.446
LIHC	cg20550118	CRABP1	0.144	0.006	-0.002	0.969	0.321
LIHC	cg25925945	NCKAP1	0.182	0	0.156	0.003	1.005

LIHC	cg26372517	TFAP2E	-0.235	0	-0.209	0	-2.118
LUAD	cg00727310	IRS1	-0.544	0	-0.537	0	-35.099
LUAD	cg02499843	PLXNA4	-0.097	0.047	-0.029	0.549	-0.136
LUAD	cg03486383	SLC7A5	0.047	0.333	0.05	0.304	0.049
LUAD	cg04218899	LAMC2	0.037	0.449	0.119	0.015	0.23
LUAD	cg05486213	MPPED2	-0.097	0.046	-0.033	0.496	-0.141
LUAD	cg06458239	ZNF549	-0.625	0	-0.545	0	-46.979
LUAD	cg07604651	FEZF1	-0.108	0.027	-0.091	0.061	-0.279
LUAD	cg10944833	NUP153	0.038	0.437	0.004	0.941	0.014
LUAD	cg11476211	PRKCE	-0.136	0.005	-0.096	0.049	-0.437
LUAD	cg11932564	TNFRSF13C	-0.146	0.003	-0.05	0.31	-0.401
LUAD	cg16393928	PRDM16	-0.264	0	-0.223	0	-3.164
LUAD	cg18397523	EVX1	-0.007	0.889	0.048	0.33	0.023
LUAD	cg18930863	DSCAML1	0.123	0.012	0.135	0.006	0.543
LUAD	cg20014398	PAX6	0.068	0.162	-0.162	0.001	-0.443
LUAD	cg20550118	CRABP1	-0.14	0.004	-0.152	0.002	-0.752
LUAD	cg25925945	NCKAP1	0.034	0.488	0.081	0.097	0.093
LUAD	cg26372517	TFAP2E	-0.143	0.003	-0.054	0.267	-0.384
LUSC	cg00727310	IRS1	-0.47	0	-0.486	0	-19.942
LUSC	cg02499843	PLXNA4	0.088	0.097	0.153	0.004	0.459
LUSC	cg03486383	SLC7A5	0.116	0.029	0.039	0.468	0.192
LUSC	cg04218899	LAMC2	-0.098	0.067	0.034	0.529	-0.105
LUSC	cg05486213	MPPED2	-0.149	0.005	-0.174	0.001	-0.86
LUSC	cg06458239	ZNF549	-0.685	0	-0.685	0	-67.73
LUSC	cg07604651	FEZF1	-0.163	0.002	-0.248	0	-1.831
LUSC	cg10944833	NUP153	0.053	0.323	0.041	0.446	0.04
LUSC	cg11476211	PRKCE	-0.119	0.026	-0.055	0.301	-0.217
LUSC	cg11932564	TNFRSF13C	-0.078	0.145	0.014	0.796	-0.064
LUSC	cg16393928	PRDM16	0.193	0	0.249	0	2.1
LUSC	cg18397523	EVX1	0.059	0.268	0.234	0	1.219
LUSC	cg18930863	DSCAML1	0.111	0.037	0.168	0.002	0.63
LUSC	cg20014398	PAX6	0.523	0	0.571	0	31.162
LUSC	cg20550118	CRABP1	0.178	0.001	0.134	0.012	0.809
LUSC	cg25925945	NCKAP1	-0.044	0.414	0.002	0.972	-0.017
LUSC	cg26372517	TFAP2E	-0.086	0.105	-0.026	0.629	-0.09
MESO	cg00727310	IRS1	-0.32	0.004	-0.229	0.041	-1.093
MESO	cg02499843	PLXNA4	0.012	0.917	-0.017	0.884	0
MESO	cg03486383	SLC7A5	0.12	0.29	0.003	0.976	0.064
MESO	cg04218899	LAMC2	-0.271	0.015	-0.099	0.383	-0.537
MESO	cg05486213	MPPED2	-0.196	0.081	-0.176	0.118	-0.378
MESO	cg06458239	ZNF549	-0.659	0	-0.635	0	-13.021
MESO	cg07604651	FEZF1	0.006	0.961	0.033	0.768	0.004
MESO	cg10944833	NUP153	-0.113	0.318	-0.057	0.615	-0.068
MESO	cg11476211	PRKCE	0.083	0.463	0.086	0.448	0.058
MESO	cg11932564	TNFRSF13C	-0.098	0.385	0.193	0.086	0.166
MESO	cg16393928	PRDM16	0.185	0.1	0.385	0	1.489
MESO	cg18397523	EVX1	0.107	0.347	0.104	0.358	0.095
MESO	cg18930863	DSCAML1	0.127	0.263	0.126	0.266	0.146
MESO	cg20014398	PAX6	0.452	0	0.544	0	5.732
MESO	cg20550118	CRABP1	-0.017	0.88	0.087	0.44	0.03
MESO	cg25925945	NCKAP1	-0.1	0.378	0.015	0.892	-0.041
MESO	cg26372517	TFAP2E	0.164	0.147	0.217	0.053	0.413
PAAD	cg00727310	IRS1	-0.573	0	-0.6	0	-19.981
PAAD	cg02499843	PLXNA4	-0.208	0.005	-0.198	0.008	-0.885
PAAD	cg03486383	SLC7A5	-0.053	0.487	-0.044	0.562	-0.027
PAAD	cg04218899	LAMC2	-0.185	0.014	-0.15	0.046	-0.546
PAAD	cg05486213	MPPED2	-0.245	0.001	-0.289	0	-1.889
PAAD	cg06458239	ZNF549	-0.747	0	-0.719	0	-44.632
PAAD	cg07604651	FEZF1	-0.102	0.177	-0.097	0.199	-0.145
PAAD	cg10944833	NUP153	-0.316	0	-0.306	0	-2.862
PAAD	cg11476211	PRKCE	-0.125	0.098	0.026	0.736	-0.122
PAAD	cg11932564	TNFRSF13C	-0.21	0.005	-0.196	0.009	-0.886
PAAD	cg16393928	PRDM16	-0.141	0.061	-0.099	0.189	-0.243
PAAD	cg18397523	EVX1	-0.053	0.484	0.224	0.003	0.556
PAAD	cg18930863	DSCAML1	0.179	0.017	0.153	0.042	0.525
PAAD	cg20014398	PAX6	-0.428	0	-0.319	0	-5.199
PAAD	cg20550118	CRABP1	0.001	0.992	0.166	0.027	0.26

PAAD	cg25925945	NCKAP1	0.032	0.675	0.108	0.154	0.093
PCPG	cg00727310	IRS1	-0.5	0	-0.325	0	-7.482
PCPG	cg02499843	PLXNA4	-0.071	0.352	-0.085	0.262	-0.082
PCPG	cg03486383	SLC7A5	0.016	0.836	-0.03	0.69	-0.004
PCPG	cg04218899	LAMC2	-0.151	0.046	-0.236	0.002	-0.861
PCPG	cg05486213	MPPED2	-0.073	0.339	-0.097	0.201	-0.102
PCPG	cg06458239	ZNF549	-0.11	0.147	-0.183	0.016	-0.421
PCPG	cg07604651	FEZF1	0.052	0.495	0.019	0.798	0.018
PCPG	cg10944833	NUP153	0.059	0.441	0.08	0.295	0.063
PCPG	cg11476211	PRKCE	-0.189	0.012	-0.082	0.279	-0.405
PCPG	cg11932564	TNFRSF13C	0.123	0.105	0.122	0.107	0.24
PCPG	cg16393928	PRDM16	0.033	0.665	0.186	0.014	0.354
PCPG	cg18397523	EVX1	-0.051	0.507	0.064	0.401	0.01
PCPG	cg18930863	DSCAML1	-0.259	0.001	-0.192	0.011	-1.224
PCPG	cg20014398	PAX6	0.185	0.014	-0.065	0.395	0.317
PCPG	cg20550118	CRABP1	0.071	0.351	0.114	0.133	0.132
PCPG	cg25925945	NCKAP1	0.006	0.942	-0.004	0.953	0
PCPG	cg26372517	TFAP2E	0.365	0	0.25	0.001	3.027
PRAD	cg00727310	IRS1	-0.437	0	-0.456	0	-21.161
PRAD	cg02499843	PLXNA4	-0.002	0.957	0.031	0.507	0.009
PRAD	cg03486383	SLC7A5	-0.016	0.737	-0.013	0.773	-0.004
PRAD	cg04218899	LAMC2	-0.322	0	-0.225	0	-5.251
PRAD	cg05486213	MPPED2	0.079	0.087	0.08	0.083	0.17
PRAD	cg06458239	ZNF549	-0.12	0.009	-0.109	0.018	-0.435
PRAD	cg07604651	FEZF1	-0.046	0.316	-0.052	0.264	-0.053
PRAD	cg10944833	NUP153	-0.195	0	-0.191	0	-1.778
PRAD	cg11476211	PRKCE	-0.242	0	-0.219	0	-2.956
PRAD	cg11932564	TNFRSF13C	0.019	0.674	-0.018	0.691	0
PRAD	cg16393928	PRDM16	0.309	0	0.373	0	9.475
PRAD	cg18397523	EVX1	-0.503	0	-0.521	0	-32.83
PRAD	cg18930863	DSCAML1	0.127	0.006	0.19	0	1.136
PRAD	cg20014398	PAX6	-0.127	0.006	-0.132	0.004	-0.602
PRAD	cg20550118	CRABP1	0.091	0.048	0.091	0.049	0.24
PRAD	cg25925945	NCKAP1	0.13	0.005	0.157	0.001	0.803
PRAD	cg26372517	TFAP2E	0.017	0.715	0.019	0.682	0.006
READ	cg00727310	IRS1	-0.315	0.002	-0.261	0.011	-1.362
READ	cg02499843	PLXNA4	0.054	0.604	0.003	0.973	0.012
READ	cg03486383	SLC7A5	-0.053	0.61	0.031	0.767	-0.008
READ	cg04218899	LAMC2	0.083	0.428	0.099	0.342	0.077
READ	cg05486213	MPPED2	-0.102	0.328	0.016	0.875	-0.048
READ	cg06458239	ZNF549	-0.834	0	-0.829	0	-40.776
READ	cg07604651	FEZF1	-0.102	0.327	-0.001	0.995	-0.05
READ	cg10944833	NUP153	-0.068	0.514	-0.054	0.602	-0.032
READ	cg11476211	PRKCE	-0.066	0.525	-0.239	0.021	-0.421
READ	cg11932564	TNFRSF13C	0.001	0.993	0.073	0.484	0.023
READ	cg16393928	PRDM16	0.193	0.062	0.175	0.092	0.414
READ	cg18397523	EVX1	0.003	0.974	-0.184	0.076	-0.205
READ	cg18930863	DSCAML1	0.126	0.227	0.162	0.118	0.231
READ	cg20014398	PAX6	0.255	0.013	0.294	0.004	1.186
READ	cg20550118	CRABP1	-0.073	0.484	-0.26	0.011	-0.528
READ	cg25925945	NCKAP1	0.05	0.631	0.131	0.209	0.099
READ	cg26372517	TFAP2E	-0.024	0.815	0.143	0.17	0.108
SKCM	cg00727310	IRS1	-0.134	0.176	-0.039	0.696	-0.107
SKCM	cg02499843	PLXNA4	-0.104	0.297	-0.12	0.227	-0.132
SKCM	cg03486383	SLC7A5	-0.278	0.005	-0.365	0	-2.051
SKCM	cg04218899	LAMC2	-0.39	0	-0.329	0.001	-2.729
SKCM	cg05486213	MPPED2	-0.242	0.014	-0.268	0.006	-1.042
SKCM	cg06458239	ZNF549	-0.312	0.001	-0.247	0.012	-1.374
SKCM	cg07604651	FEZF1	-0.097	0.329	0.008	0.939	-0.047
SKCM	cg10944833	NUP153	0.188	0.057	0.195	0.049	0.488
SKCM	cg11476211	PRKCE	-0.155	0.119	-0.117	0.239	-0.216
SKCM	cg11932564	TNFRSF13C	0.148	0.136	-0.003	0.976	0.128
SKCM	cg16393928	PRDM16	-0.052	0.601	-0.032	0.747	-0.016
SKCM	cg18930863	DSCAML1	0.086	0.386	-0.001	0.992	0.036
SKCM	cg20014398	PAX6	0.258	0.008	0.355	0	1.821
SKCM	cg20550118	CRABP1	-0.091	0.362	-0.158	0.111	-0.191
SKCM	cg25925945	NCKAP1	-0.079	0.428	-0.008	0.933	-0.029

SKCM	cg26372517	TFAP2E	-0.134	0.178	-0.144	0.146	-0.221
STAD	cg00727310	IRS1	-0.259	0	-0.313	0	-4.029
STAD	cg02499843	PLXNA4	-0.218	0	-0.278	0	-2.704
STAD	cg03486383	SLC7A5	-0.122	0.028	-0.098	0.078	-0.299
STAD	cg04218899	LAMC2	-0.311	0	-0.159	0.004	-2.894
STAD	cg05486213	MPPED2	-0.232	0	-0.254	0	-2.467
STAD	cg06458239	ZNF549	-0.767	0	-0.803	0	-108.544
STAD	cg07604651	FEZF1	-0.222	0	-0.154	0.005	-1.302
STAD	cg10944833	NUP153	-0.06	0.278	-0.075	0.175	-0.09
STAD	cg11476211	PRKCE	-0.239	0	-0.247	0	-2.459
STAD	cg11932564	TNFRSF13C	-0.09	0.103	-0.036	0.519	-0.099
STAD	cg16393928	PRDM16	-0.232	0	-0.235	0	-2.201
STAD	cg18397523	EVX1	-0.269	0	-0.209	0	-2.445
STAD	cg18930863	DSCAML1	0.116	0.036	0.15	0.006	0.498
STAD	cg20014398	PAX6	0.27	0	0.258	0	3.119
STAD	cg20550118	CRABP1	-0.149	0.007	-0.221	0	-1.259
STAD	cg25925945	NCKAP1	-0.324	0	-0.152	0.006	-3.148
STAD	cg26372517	TFAP2E	-0.222	0	-0.205	0	-1.716
TGCT	cg00727310	IRS1	0.156	0.073	0.072	0.409	0.205
TGCT	cg02499843	PLXNA4	-0.098	0.261	-0.127	0.146	-0.163
TGCT	cg03486383	SLC7A5	0.268	0.002	0.243	0.005	1.294
TGCT	cg04218899	LAMC2	-0.14	0.108	-0.091	0.295	-0.184
TGCT	cg05486213	MPPED2	-0.257	0.003	-0.258	0.003	-1.316
TGCT	cg06458239	ZNF549	-0.306	0	-0.387	0	-3.149
TGCT	cg07604651	FEZF1	0.004	0.967	-0.118	0.175	-0.089
TGCT	cg10944833	NUP153	-0.075	0.392	-0.226	0.009	-0.495
TGCT	cg11476211	PRKCE	-0.065	0.456	0.029	0.737	-0.018
TGCT	cg11932564	TNFRSF13C	0.066	0.448	0.024	0.787	0.026
TGCT	cg16393928	PRDM16	-0.163	0.061	0.169	0.051	0.02
TGCT	cg18397523	EVX1	-0.277	0.001	-0.257	0.003	-1.466
TGCT	cg18930863	DSCAML1	0.582	0	0.689	0	20.722
TGCT	cg20014398	PAX6	0.516	0	0.353	0	6.593
TGCT	cg20550118	CRABP1	0.399	0	0.448	0	5.507
TGCT	cg25925945	NCKAP1	0.148	0.088	0.1	0.252	0.216
TGCT	cg26372517	TFAP2E	-0.104	0.233	-0.088	0.315	-0.11
THCA	cg00727310	IRS1	-0.193	0	-0.19	0	-1.816
THCA	cg02499843	PLXNA4	-0.144	0.001	-0.086	0.057	-0.519
THCA	cg03486383	SLC7A5	0.071	0.116	0	0.991	0.066
THCA	cg04218899	LAMC2	0.009	0.842	-0.037	0.406	-0.014
THCA	cg05486213	MPPED2	-0.104	0.02	-0.143	0.001	-0.586
THCA	cg06458239	ZNF549	-0.046	0.311	-0.017	0.705	-0.026
THCA	cg10944833	NUP153	-0.074	0.101	-0.049	0.274	-0.101
THCA	cg11476211	PRKCE	-0.047	0.292	0.023	0.609	-0.02
THCA	cg11932564	TNFRSF13C	-0.165	0	-0.26	0	-2.768
THCA	cg16393928	PRDM16	-0.077	0.086	-0.174	0	-0.782
THCA	cg18397523	EVX1	0.043	0.34	0.055	0.222	0.056
THCA	cg18930863	DSCAML1	0.054	0.234	0.053	0.238	0.067
THCA	cg20014398	PAX6	-0.089	0.047	-0.065	0.149	-0.172
THCA	cg20550118	CRABP1	-0.02	0.662	0.049	0.271	0.025
THCA	cg25925945	NCKAP1	0.017	0.702	0.041	0.365	0.021
THCA	cg26372517	TFAP2E	0.009	0.847	0.041	0.357	0.019
THYM	cg00727310	IRS1	-0.278	0.002	-0.274	0.003	-1.444
THYM	cg02499843	PLXNA4	-0.082	0.379	-0.002	0.982	-0.034
THYM	cg03486383	SLC7A5	0.166	0.072	0.132	0.154	0.297
THYM	cg04218899	LAMC2	-0.575	0	-0.609	0	-13.983
THYM	cg05486213	MPPED2	-0.246	0.007	-0.308	0.001	-1.494
THYM	cg06458239	ZNF549	-0.367	0	-0.285	0.002	-2.386
THYM	cg07604651	FEZF1	0.156	0.091	0.212	0.021	0.52
THYM	cg10944833	NUP153	-0.019	0.841	-0.146	0.114	-0.14
THYM	cg11476211	PRKCE	-0.187	0.043	-0.047	0.614	-0.265
THYM	cg11932564	TNFRSF13C	-0.023	0.806	0.046	0.621	0.007
THYM	cg16393928	PRDM16	0.157	0.09	-0.003	0.975	0.163
THYM	cg18397523	EVX1	0.235	0.01	0.155	0.094	0.626
THYM	cg18930863	DSCAML1	-0.133	0.152	-0.237	0.01	-0.585
THYM	cg20014398	PAX6	0.434	0	0.081	0.385	2.66
THYM	cg20550118	CRABP1	0.098	0.291	0.33	0	1.235

THYM	cg25925945	NCKAP1	-0.128	0.168	-0.108	0.245	-0.165
THYM	cg26372517	TFAP2E	0.163	0.077	0.139	0.133	0.304
UCEC	cg00727310	IRS1	-0.428	0	-0.44	0	-17.284
UCEC	cg02499843	PLXNA4	-0.129	0.008	-0.117	0.017	-0.474
UCEC	cg03486383	SLC7A5	0.118	0.016	-0.013	0.784	0.209
UCEC	cg04218899	LAMC2	-0.353	0	-0.11	0.024	-4.77
UCEC	cg05486213	MPPED2	-0.195	0	-0.159	0.001	-1.297
UCEC	cg06458239	ZNF549	-0.585	0	-0.471	0	-33.992
UCEC	cg10944833	NUP153	0.04	0.41	0.06	0.221	0.055
UCEC	cg11476211	PRKCE	-0.215	0	-0.246	0	-2.668
UCEC	cg11932564	TNFRSF13C	0.088	0.074	-0.014	0.775	0.097
UCEC	cg16393928	PRDM16	-0.005	0.921	0.108	0.027	0.168
UCEC	cg18397523	EVX1	-0.124	0.011	-0.066	0.175	-0.292
UCEC	cg18930863	DSCAML1	0.11	0.025	0.179	0	0.821
UCEC	cg20550118	CRABP1	0.051	0.3	-0.049	0.314	0.002
UCEC	cg25925945	NCKAP1	-0.068	0.163	-0.064	0.189	-0.101
UCEC	cg26372517	TFAP2E	-0.071	0.146	-0.05	0.313	-0.085
UCS	cg00727310	IRS1	-0.499	0	-0.478	0	-3.734
UCS	cg02499843	PLXNA4	-0.245	0.071	-0.279	0.039	-0.672
UCS	cg03486383	SLC7A5	-0.27	0.046	-0.256	0.059	-0.676
UCS	cg04218899	LAMC2	-0.348	0.009	-0.278	0.04	-1.099
UCS	cg05486213	MPPED2	-0.163	0.235	-0.117	0.394	-0.15
UCS	cg06458239	ZNF549	-0.629	0	-0.621	0	-8.097
UCS	cg07604651	FEZF1	-0.282	0.037	-0.252	0.063	-0.706
UCS	cg10944833	NUP153	0.07	0.612	0.11	0.424	0.056
UCS	cg11476211	PRKCE	-0.202	0.138	-0.029	0.831	-0.176
UCS	cg11932564	TNFRSF13C	-0.196	0.151	-0.32	0.017	-0.724
UCS	cg16393928	PRDM16	0.298	0.027	0.285	0.035	0.882
UCS	cg18397523	EVX1	-0.082	0.554	0.014	0.918	-0.02
UCS	cg18930863	DSCAML1	-0.225	0.098	-0.117	0.395	-0.274
UCS	cg20014398	PAX6	0.501	0	0.639	0	6.374
UCS	cg20550118	CRABP1	-0.247	0.069	-0.16	0.245	-0.383
UCS	cg25925945	NCKAP1	-0.096	0.485	-0.157	0.252	-0.124
UCS	cg26372517	TFAP2E	0.003	0.983	-0.052	0.708	-0.008
UVM	cg00727310	IRS1	-0.224	0.05	-0.198	0.084	-0.505
UVM	cg02499843	PLXNA4	0.082	0.476	0.042	0.714	0.033
UVM	cg03486383	SLC7A5	-0.37	0.001	-0.531	0	-4.401
UVM	cg04218899	LAMC2	-0.081	0.486	0.107	0.352	0.023
UVM	cg05486213	MPPED2	-0.316	0.005	-0.272	0.017	-1.21
UVM	cg06458239	ZNF549	-0.16	0.164	-0.207	0.07	-0.365
UVM	cg07604651	FEZF1	-0.073	0.53	-0.089	0.44	-0.052
UVM	cg10944833	NUP153	-0.086	0.456	-0.042	0.72	-0.035
UVM	cg11476211	PRKCE	-0.022	0.85	-0.12	0.299	-0.064
UVM	cg11932564	TNFRSF13C	-0.039	0.737	0.077	0.504	0.018
UVM	cg16393928	PRDM16	0.409	0	0.496	0	4.15
UVM	cg18397523	EVX1	-0.133	0.25	-0.175	0.127	-0.237
UVM	cg18930863	DSCAML1	0.065	0.575	0.079	0.496	0.04
UVM	cg20014398	PAX6	-0.125	0.277	-0.056	0.626	-0.081
UVM	cg20550118	CRABP1	-0.03	0.793	-0.003	0.977	-0.003
UVM	cg25925945	NCKAP1	-0.025	0.826	-0.014	0.906	-0.003
UVM	cg26372517	TFAP2E	0.347	0.002	0.177	0.123	1.097

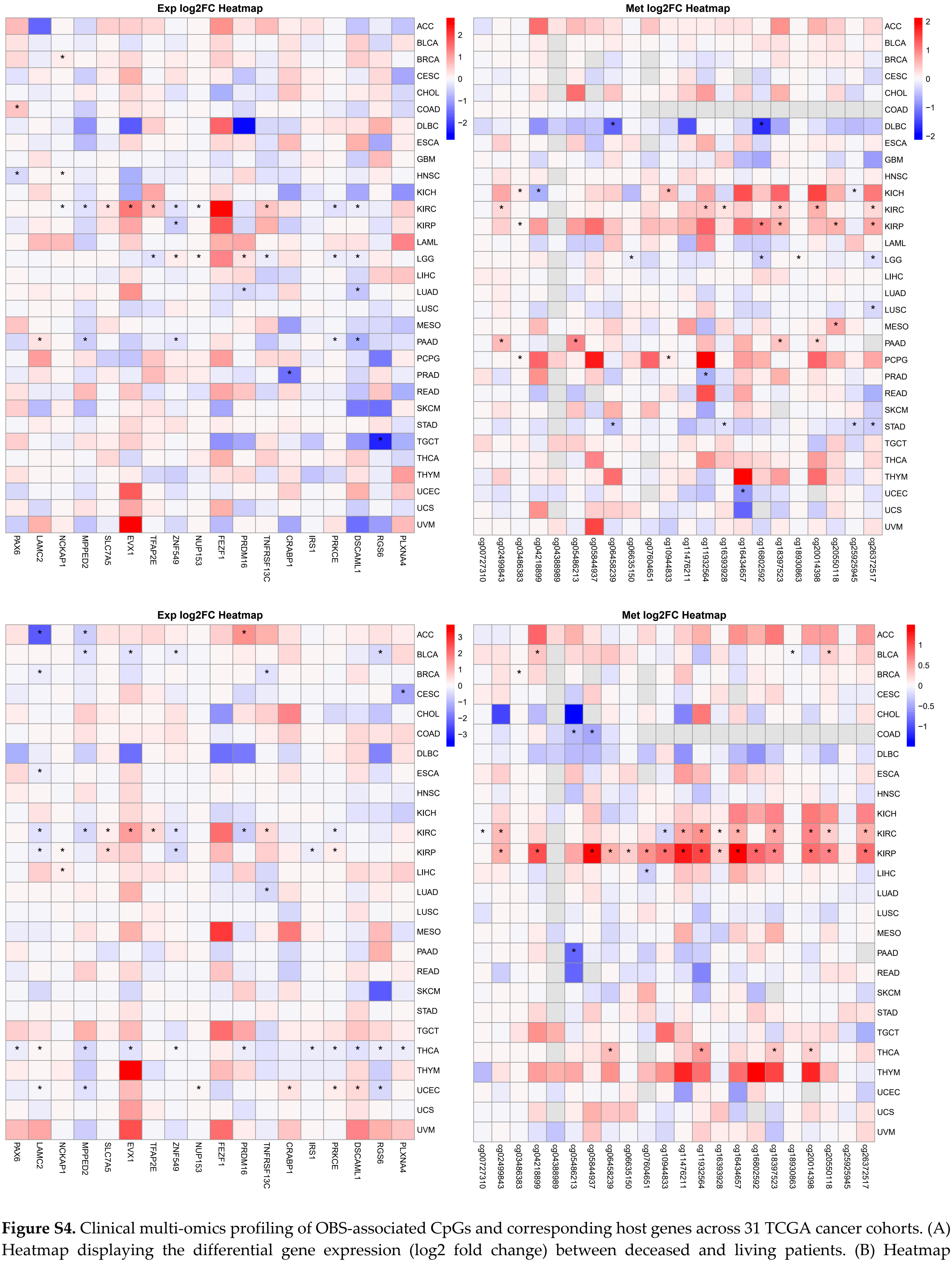

Figure S4. Clinical multi-omics profiling of OBS-associated CpGs and corresponding host genes across 31 TCGA cancer cohorts. (A) Heatmap displaying the differential gene expression (log2 fold change) between deceased and living patients. (B) Heatmap displaying the differential DNA methylation (log2 fold change) between deceased and living patients. (C) Heatmap displaying the differential gene expression (log2 fold change) between advanced (Stage III/IV) and early (Stage I/II) tumor stages. (D) Heatmap displaying the differential DNA methylation (log2 fold change) between advanced and early tumor stages. Red cells indicate upregulated expression or hypermethylation in the clinically severe groups (deceased or late-stage), whereas blue cells indicate downregulated expression or hypomethylation. Asterisks (*) denote statistically significant differences (FDR < 0.05).

Table S2. Cross-species age-related methylation correlation coefficients for the 26 human OBS-associated CpG sites. The table presents the Pearson and Spearman correlation coefficients calculated between chronological age and methylation Beta-values for each of the 26 prioritized CpG loci across nine mammalian species (e.g., *Macaca mulatta, Mus musculus*, etc.). Data was derived from the Mammalian Methylation Consortium dataset (GSE223748).

ID	Species	Pearson	Spearman	Sex	Chr.	Location	Gene symbol	Genomic loc.	CpG island
cg00727310	*Canis lupus familiaris*	-0.18	-0.17	M	2	227662704	IRS1	1stExon	Island
cg00727310	*Capreolus capreolus*	0.26	0.21	M	2	227662704	IRS1	1stExon	Island
cg00727310	*Equus caballus*	0.3	0.3	M	2	227662704	IRS1	1stExon	Island
cg00727310	*Felis catus*	-0.2	-0.31	M	2	227662704	IRS1	1stExon	Island
cg00727310	*Homo sapiens*	0.06	0.01	M	2	227662704	IRS1	1stExon	Island
cg00727310	*Macaca mulatta*	-0.35	-0.36	M	2	227662704	IRS1	1stExon	Island
cg00727310	*Mus musculus*	-0.02	0.03	M	2	227662704	IRS1	1stExon	Island
cg00727310	*Ovis aries*	0.01	0	M	2	227662704	IRS1	1stExon	Island
cg00727310	*Rattus norvegicus*	-0.36	-0.41	M	2	227662704	IRS1	1stExon	Island
cg02132051	*Canis lupus familiaris*	0.05	0.13	M	14	73020007	RGS6	Body	
cg02132051	*Capreolus capreolus*	-0.37	-0.34	M	14	73020007	RGS6	Body	
cg02132051	*Equus caballus*	0.25	0.15	M	14	73020007	RGS6	Body	
cg02132051	*Felis catus*	0.32	0.33	M	14	73020007	RGS6	Body	
cg02132051	*Homo sapiens*	-0.28	-0.3	M	14	73020007	RGS6	Body	
cg02132051	*Macaca mulatta*	-0.4	-0.36	M	14	73020007	RGS6	Body	
cg02132051	*Mus musculus*	-0.78	-0.81	M	14	73020007	RGS6	Body	
cg02132051	*Ovis aries*	-0.2	-0.17	M	14	73020007	RGS6	Body	
cg02132051	*Rattus norvegicus*	0.31	0.27	M	14	73020007	RGS6	Body	
cg02499843	*Canis lupus familiaris*	-0.07	-0.07	M	7	132261287	PLXNA4	1stExon	Island
cg02499843	*Capreolus capreolus*	-0.2	-0.25	M	7	132261287	PLXNA4	1stExon	Island
cg02499843	*Equus caballus*	0.71	0.72	M	7	132261287	PLXNA4	1stExon	Island
cg02499843	*Felis catus*	0.19	0.21	M	7	132261287	PLXNA4	1stExon	Island
cg02499843	*Homo sapiens*	-0.28	-0.32	M	7	132261287	PLXNA4	1stExon	Island
cg02499843	*Macaca mulatta*	0.34	0.44	M	7	132261287	PLXNA4	1stExon	Island
cg02499843	*Mus musculus*	0.45	0.47	M	7	132261287	PLXNA4	1stExon	Island
cg02499843	*Ovis aries*	-0.06	-0.1	M	7	132261287	PLXNA4	1stExon	Island
cg02499843	*Rattus norvegicus*	0.23	0.21	M	7	132261287	PLXNA4	1stExon	Island
cg02650266	*Canis lupus familiaris*	0.01	0.04	F	4	147558239			Island
cg02650266	*Capreolus capreolus*	-0.3	-0.28	F	4	147558239			Island
cg02650266	*Equus caballus*	0	-0.03	F	4	147558239			Island
cg02650266	*Felis catus*	0.17	0.09	F	4	147558239			Island
cg02650266	*Homo sapiens*	0.67	0.72	F	4	147558239			Island
cg02650266	*Macaca mulatta*	0.53	0.61	F	4	147558239			Island
cg02650266	*Mus musculus*	-0.41	-0.43	F	4	147558239			Island
cg02650266	*Ovis aries*	-0.08	-0.04	F	4	147558239			Island
cg02650266	*Rattus norvegicus*	0.63	0.67	F	4	147558239			Island
cg03486383	*Canis lupus familiaris*	-0.02	-0.02	M	16	87865062	SLC7A5	3'UTR	
cg03486383	*Capreolus capreolus*	-0.05	-0.09	M	16	87865062	SLC7A5	3'UTR	
cg03486383	*Equus caballus*	-0.19	-0.22	M	16	87865062	SLC7A5	3'UTR	
cg03486383	*Felis catus*	-0.19	-0.18	M	16	87865062	SLC7A5	3'UTR	
cg03486383	*Homo sapiens*	-0.24	-0.25	M	16	87865062	SLC7A5	3'UTR	
cg03486383	*Macaca mulatta*	0.01	-0.12	M	16	87865062	SLC7A5	3'UTR	
cg03486383	*Mus musculus*	-0.01	0.02	M	16	87865062	SLC7A5	3'UTR	
cg03486383	*Ovis aries*	-0.04	-0.09	M	16	87865062	SLC7A5	3'UTR	
cg03486383	*Rattus norvegicus*	0.21	0.23	M	16	87865062	SLC7A5	3'UTR	
cg03846926	*Canis lupus familiaris*	0.33	0.37	F	10	21807211	C10orf140	5'UTR	S_Shore
cg03846926	*Capreolus capreolus*	0.5	0.53	F	10	21807211	C10orf140	5'UTR	S_Shore
cg03846926	*Equus caballus*	0.27	0.31	F	10	21807211	C10orf140	5'UTR	S_Shore
cg03846926	*Felis catus*	0.04	0.08	F	10	21807211	C10orf140	5'UTR	S_Shore
cg03846926	*Homo sapiens*	-0.42	-0.68	F	10	21807211	C10orf140	5'UTR	S_Shore
cg03846926	*Macaca mulatta*	0.04	-0.01	F	10	21807211	C10orf140	5'UTR	S_Shore
cg03846926	*Mus musculus*	0.34	0.37	F	10	21807211	C10orf140	5'UTR	S_Shore
cg03846926	*Ovis aries*	0.24	0.17	F	10	21807211	C10orf140	5'UTR	S_Shore
cg03846926	*Rattus norvegicus*	-0.27	-0.35	F	10	21807211	C10orf140	5'UTR	S_Shore
cg04218899	*Canis lupus familiaris*	0.1	0.1	F	1	183155366	LAMC2	1stExon	
cg04218899	*Capreolus capreolus*	0.62	0.76	F	1	183155366	LAMC2	1stExon	
cg04218899	*Equus caballus*	0.26	0.24	F	1	183155366	LAMC2	1stExon	
cg04218899	*Felis catus*	0.24	0.29	F	1	183155366	LAMC2	1stExon	
cg04218899	*Homo sapiens*	-0.1	-0.05	F	1	183155366	LAMC2	1stExon	

cg04218899	*Macaca mulatta*	0.3	0.33	F	1	183155366	LAMC2	1stExon	
cg04218899	*Mus musculus*	0.17	0.1	F	1	183155366	LAMC2	1stExon	
cg04218899	*Ovis aries*	-0.18	-0.11	F	1	183155366	LAMC2	1stExon	
cg04218899	*Rattus norvegicus*	0.35	0.47	F	1	183155366	LAMC2	1stExon	
cg04388989	*Canis lupus familiaris*	0.79	0.84	M	19	30715606			Island
cg04388989	*Capreolus capreolus*	0.76	0.81	M	19	30715606			Island
cg04388989	*Equus caballus*	0.72	0.7	M	19	30715606			Island
cg04388989	*Felis catus*	0.61	0.68	M	19	30715606			Island
cg04388989	*Homo sapiens*	0.2	0.3	M	19	30715606			Island
cg04388989	*Macaca mulatta*	0.28	0.32	M	19	30715606			Island
cg04388989	*Mus musculus*	-0.4	-0.39	M	19	30715606			Island
cg04388989	*Ovis aries*	0.42	0.45	M	19	30715606			Island
cg04388989	*Rattus norvegicus*	0.05	-0.08	M	19	30715606			Island
cg05486213	*Canis lupus familiaris*	0.15	0.17	M	11	30607950	MPPED2	TSS200	Island
cg05486213	*Capreolus capreolus*	0.84	0.86	M	11	30607950	MPPED2	TSS200	Island
cg05486213	*Equus caballus*	0.72	0.7	M	11	30607950	MPPED2	TSS200	Island
cg05486213	*Felis catus*	0.38	0.41	M	11	30607950	MPPED2	TSS200	Island
cg05486213	*Homo sapiens*	-0.16	-0.2	M	11	30607950	MPPED2	TSS200	Island
cg05486213	*Macaca mulatta*	0.36	0.42	M	11	30607950	MPPED2	TSS200	Island
cg05486213	*Mus musculus*	-0.38	-0.39	M	11	30607950	MPPED2	TSS200	Island
cg05486213	*Ovis aries*	0.23	0.25	M	11	30607950	MPPED2	TSS200	Island
cg05486213	*Rattus norvegicus*	0.33	0.41	M	11	30607950	MPPED2	TSS200	Island
cg05844937	*Canis lupus familiaris*	0.25	0.27	M	6	1614141			Island
cg05844937	*Capreolus capreolus*	-0.37	-0.4	M	6	1614141			Island
cg05844937	*Equus caballus*	0.68	0.68	M	6	1614141			Island
cg05844937	*Felis catus*	0.4	0.4	M	6	1614141			Island
cg05844937	*Homo sapiens*	-0.48	-0.57	M	6	1614141			Island
cg05844937	*Macaca mulatta*	0.12	0.23	M	6	1614141			Island
cg05844937	*Mus musculus*	-0.14	-0.09	M	6	1614141			Island
cg05844937	*Ovis aries*	-0.09	-0.08	M	6	1614141			Island
cg05844937	*Rattus norvegicus*	0.53	0.5	M	6	1614141			Island
cg06458239	*Canis lupus familiaris*	0.04	0.02	F	19	58038573	ZNF549	TSS200	Island
cg06458239	*Capreolus capreolus*	0.09	0.12	F	19	58038573	ZNF549	TSS200	Island
cg06458239	*Equus caballus*	0.01	0.02	F	19	58038573	ZNF549	TSS200	Island
cg06458239	*Felis catus*	0.12	0.19	F	19	58038573	ZNF549	TSS200	Island
cg06458239	*Homo sapiens*	-0.19	-0.11	F	19	58038573	ZNF549	TSS200	Island
cg06458239	*Macaca mulatta*	0.04	0.04	F	19	58038573	ZNF549	TSS200	Island
cg06458239	*Mus musculus*	-0.1	-0.05	F	19	58038573	ZNF549	TSS200	Island
cg06458239	*Ovis aries*	0.26	0.23	F	19	58038573	ZNF549	TSS200	Island
cg06458239	*Rattus norvegicus*	0.13	0.19	F	19	58038573	ZNF549	TSS200	Island
cg06635150	*Canis lupus familiaris*	-0.29	-0.31	F	5	171094937			
cg06635150	*Capreolus capreolus*	-0.11	-0.04	F	5	171094937			
cg06635150	*Equus caballus*	-0.2	-0.21	F	5	171094937			
cg06635150	*Felis catus*	-0.02	0.06	F	5	171094937			
cg06635150	*Homo sapiens*	0.18	0.12	F	5	171094937			
cg06635150	*Macaca mulatta*	-0.1	-0.12	F	5	171094937			
cg06635150	*Mus musculus*	-0.12	-0.18	F	5	171094937			
cg06635150	*Ovis aries*	0.09	-0.03	F	5	171094937			
cg06635150	*Rattus norvegicus*	0.42	0.41	F	5	171094937			
cg07604651	*Canis lupus familiaris*	0.04	0.03	M	7	121944755	FEZF1	TSS200	N_Shore
cg07604651	*Capreolus capreolus*	0.71	0.78	M	7	121944755	FEZF1	TSS200	N_Shore
cg07604651	*Equus caballus*	0.33	0.41	M	7	121944755	FEZF1	TSS200	N_Shore
cg07604651	*Felis catus*	0.82	0.87	M	7	121944755	FEZF1	TSS200	N_Shore
cg07604651	*Homo sapiens*	-0.45	-0.63	M	7	121944755	FEZF1	TSS200	N_Shore
cg07604651	*Macaca mulatta*	0.09	0.11	M	7	121944755	FEZF1	TSS200	N_Shore
cg07604651	*Mus musculus*	0.44	0.53	M	7	121944755	FEZF1	TSS200	N_Shore
cg07604651	*Ovis aries*	0.17	0.16	M	7	121944755	FEZF1	TSS200	N_Shore
cg07604651	*Rattus norvegicus*	0.76	0.81	M	7	121944755	FEZF1	TSS200	N_Shore
cg10944833	*Canis lupus familiaris*	0	-0.02	F	6	17702460	NUP153	Body	N_Shelf
cg10944833	*Capreolus capreolus*	0.18	0.23	F	6	17702460	NUP153	Body	N_Shelf
cg10944833	*Equus caballus*	0.19	0.2	F	6	17702460	NUP153	Body	N_Shelf
cg10944833	*Felis catus*	0.2	0.1	F	6	17702460	NUP153	Body	N_Shelf
cg10944833	*Homo sapiens*	0.21	0.23	F	6	17702460	NUP153	Body	N_Shelf
cg10944833	*Macaca mulatta*	-0.19	-0.22	F	6	17702460	NUP153	Body	N_Shelf
cg10944833	*Mus musculus*	-0.23	-0.24	F	6	17702460	NUP153	Body	N_Shelf
cg10944833	*Ovis aries*	-0.04	-0.05	F	6	17702460	NUP153	Body	N_Shelf
cg10944833	*Rattus norvegicus*	0.05	-0.05	F	6	17702460	NUP153	Body	N_Shelf

cg11476211	*Canis lupus familiaris*	0.32	0.37	F	2	45879284	PRKCE	1stExon	Island
cg11476211	*Capreolus capreolus*	0.33	0.32	F	2	45879284	PRKCE	1stExon	Island
cg11476211	*Equus caballus*	-0.03	-0.03	F	2	45879284	PRKCE	1stExon	Island
cg11476211	*Felis catus*	-0.52	-0.47	F	2	45879284	PRKCE	1stExon	Island
cg11476211	*Homo sapiens*	-0.39	-0.4	F	2	45879284	PRKCE	1stExon	Island
cg11476211	*Macaca mulatta*	0.33	0.41	F	2	45879284	PRKCE	1stExon	Island
cg11476211	*Mus musculus*	-0.37	-0.38	F	2	45879284	PRKCE	1stExon	Island
cg11476211	*Ovis aries*	-0.18	-0.12	F	2	45879284	PRKCE	1stExon	Island
cg11476211	*Rattus norvegicus*	0.67	0.59	F	2	45879284	PRKCE	1stExon	Island
cg11932564	*Canis lupus familiaris*	0.18	0.15	M	22	42322146	TNFRSF13C	Body	Island
cg11932564	*Capreolus capreolus*	-0.34	-0.35	M	22	42322146	TNFRSF13C	Body	Island
cg11932564	*Equus caballus*	-0.01	0.06	M	22	42322146	TNFRSF13C	Body	Island
cg11932564	*Felis catus*	0	-0.08	M	22	42322146	TNFRSF13C	Body	Island
cg11932564	*Homo sapiens*	-0.09	-0.03	M	22	42322146	TNFRSF13C	Body	Island
cg11932564	*Macaca mulatta*	0.45	0.55	M	22	42322146	TNFRSF13C	Body	Island
cg11932564	*Mus musculus*	-0.42	-0.43	M	22	42322146	TNFRSF13C	Body	Island
cg11932564	*Ovis aries*	0	-0.03	M	22	42322146	TNFRSF13C	Body	Island
cg11932564	*Rattus norvegicus*	0.59	0.61	M	22	42322146	TNFRSF13C	Body	Island
cg16393928	*Canis lupus familiaris*	-0.09	-0.09	M	1	3135836	PRDM16	Body	
cg16393928	*Capreolus capreolus*	-0.25	-0.44	M	1	3135836	PRDM16	Body	
cg16393928	*Equus caballus*	-0.01	-0.19	M	1	3135836	PRDM16	Body	
cg16393928	*Felis catus*	-0.17	-0.04	M	1	3135836	PRDM16	Body	
cg16393928	*Homo sapiens*	-0.17	-0.3	M	1	3135836	PRDM16	Body	
cg16393928	*Macaca mulatta*	-0.34	-0.34	M	1	3135836	PRDM16	Body	
cg16393928	*Mus musculus*	0.04	-0.11	M	1	3135836	PRDM16	Body	
cg16393928	*Ovis aries*	-0.12	-0.09	M	1	3135836	PRDM16	Body	
cg16393928	*Rattus norvegicus*	-0.05	-0.41	M	1	3135836	PRDM16	Body	
cg16434657	*Canis lupus familiaris*	0.28	0.22	F	3	137490228			Island
cg16434657	*Capreolus capreolus*	0.55	0.77	F	3	137490228			Island
cg16434657	*Equus caballus*	0.52	0.49	F	3	137490228			Island
cg16434657	*Felis catus*	0.16	0.2	F	3	137490228			Island
cg16434657	*Homo sapiens*	-0.34	-0.29	F	3	137490228			Island
cg16434657	*Macaca mulatta*	0.14	0.19	F	3	137490228			Island
cg16434657	*Mus musculus*	0.31	0.39	F	3	137490228			Island
cg16434657	*Ovis aries*	0.32	0.14	F	3	137490228			Island
cg16434657	*Rattus norvegicus*	0.51	0.66	F	3	137490228			Island
cg16802592	*Canis lupus familiaris*	0.25	0.25	M	15	37179603	LOC145845	TSS1500	Island
cg16802592	*Capreolus capreolus*	-0.38	-0.36	M	15	37179603	LOC145845	TSS1500	Island
cg16802592	*Equus caballus*	0.5	0.52	M	15	37179603	LOC145845	TSS1500	Island
cg16802592	*Felis catus*	-0.31	-0.36	M	15	37179603	LOC145845	TSS1500	Island
cg16802592	*Homo sapiens*	0.11	0.05	M	15	37179603	LOC145845	TSS1500	Island
cg16802592	*Macaca mulatta*	-0.01	0.1	M	15	37179603	LOC145845	TSS1500	Island
cg16802592	*Mus musculus*	0.28	0.31	M	15	37179603	LOC145845	TSS1500	Island
cg16802592	*Ovis aries*	0.33	0.32	M	15	37179603	LOC145845	TSS1500	Island
cg16802592	*Rattus norvegicus*	-0.1	-0.15	M	15	37179603	LOC145845	TSS1500	Island
cg18397523	*Canis lupus familiaris*	0.63	0.71	F	7	27282351	EVX1	1stExon	Island
cg18397523	*Capreolus capreolus*	0.73	0.77	F	7	27282351	EVX1	1stExon	Island
cg18397523	*Equus caballus*	0.56	0.54	F	7	27282351	EVX1	1stExon	Island
cg18397523	*Felis catus*	0.59	0.63	F	7	27282351	EVX1	1stExon	Island
cg18397523	*Homo sapiens*	0.35	0.41	F	7	27282351	EVX1	1stExon	Island
cg18397523	*Macaca mulatta*	0.21	0.26	F	7	27282351	EVX1	1stExon	Island
cg18397523	*Mus musculus*	0.47	0.5	F	7	27282351	EVX1	1stExon	Island
cg18397523	*Ovis aries*	0.47	0.41	F	7	27282351	EVX1	1stExon	Island
cg18397523	*Rattus norvegicus*	0.11	0.58	F	7	27282351	EVX1	1stExon	Island
cg18930863	*Canis lupus familiaris*	-0.11	-0.12	M	11	117389197	DSCAML1	Body	Island
cg18930863	*Capreolus capreolus*	-0.04	0	M	11	117389197	DSCAML1	Body	Island
cg18930863	*Equus caballus*	0.16	0.08	M	11	117389197	DSCAML1	Body	Island
cg18930863	*Felis catus*	0.03	0.1	M	11	117389197	DSCAML1	Body	Island
cg18930863	*Homo sapiens*	-0.1	-0.1	M	11	117389197	DSCAML1	Body	Island
cg18930863	*Macaca mulatta*	0	0.03	M	11	117389197	DSCAML1	Body	Island
cg18930863	*Mus musculus*	-0.14	-0.12	M	11	117389197	DSCAML1	Body	Island
cg18930863	*Ovis aries*	-0.3	-0.29	M	11	117389197	DSCAML1	Body	Island
cg18930863	*Rattus norvegicus*	0.01	-0.09	M	11	117389197	DSCAML1	Body	Island
cg20014398	*Canis lupus familiaris*	0.43	0.52	F	11	31825792	PAX6	Body	Island
cg20014398	*Capreolus capreolus*	0.68	0.77	F	11	31825792	PAX6	Body	Island
cg20014398	*Equus caballus*	0.62	0.64	F	11	31825792	PAX6	Body	Island
cg20014398	*Felis catus*	0.52	0.59	F	11	31825792	PAX6	Body	Island

cg20014398	*Homo sapiens*	0.1	0.12	F	11	31825792	PAX6	Body	Island
cg20014398	*Macaca mulatta*	0.26	0.29	F	11	31825792	PAX6	Body	Island
cg20014398	*Mus musculus*	0.24	0.26	F	11	31825792	PAX6	Body	Island
cg20014398	*Ovis aries*	0.45	0.26	F	11	31825792	PAX6	Body	Island
cg20014398	*Rattus norvegicus*	0.64	0.73	F	11	31825792	PAX6	Body	Island
cg20550118	*Canis lupus familiaris*	0.1	0.1	F	15	78633662	CRABP1	Body	Island
cg20550118	*Capreolus capreolus*	-0.2	-0.2	F	15	78633662	CRABP1	Body	Island
cg20550118	*Equus caballus*	0.04	0.06	F	15	78633662	CRABP1	Body	Island
cg20550118	*Felis catus*	0.08	0.07	F	15	78633662	CRABP1	Body	Island
cg20550118	*Homo sapiens*	-0.14	-0.24	F	15	78633662	CRABP1	Body	Island
cg20550118	*Macaca mulatta*	0.16	0.17	F	15	78633662	CRABP1	Body	Island
cg20550118	*Mus musculus*	-0.11	-0.09	F	15	78633662	CRABP1	Body	Island
cg20550118	*Ovis aries*	-0.02	0.03	F	15	78633662	CRABP1	Body	Island
cg20550118	*Rattus norvegicus*	0.01	0.04	F	15	78633662	CRABP1	Body	Island
cg25925945	*Canis lupus familiaris*	0.02	-0.09	F	2	183902764	NCKAP1	1stExon	Island
cg25925945	*Capreolus capreolus*	0.8	0.87	F	2	183902764	NCKAP1	1stExon	Island
cg25925945	*Equus caballus*	0.62	0.57	F	2	183902764	NCKAP1	1stExon	Island
cg25925945	*Felis catus*	0.07	-0.04	F	2	183902764	NCKAP1	1stExon	Island
cg25925945	*Homo sapiens*	0.12	-0.03	F	2	183902764	NCKAP1	1stExon	Island
cg25925945	*Macaca mulatta*	0.34	0.41	F	2	183902764	NCKAP1	1stExon	Island
cg25925945	*Mus musculus*	0.32	0.29	F	2	183902764	NCKAP1	1stExon	Island
cg25925945	*Ovis aries*	0.23	0.22	F	2	183902764	NCKAP1	1stExon	Island
cg25925945	*Rattus norvegicus*	0.71	0.81	F	2	183902764	NCKAP1	1stExon	Island
cg26372517	*Canis lupus familiaris*	-0.02	0	F	1	36039159	TFAP2E	5'UTR	Island
cg26372517	*Capreolus capreolus*	-0.13	-0.11	F	1	36039159	TFAP2E	5'UTR	Island
cg26372517	*Equus caballus*	-0.03	0	F	1	36039159	TFAP2E	5'UTR	Island
cg26372517	*Felis catus*	0.17	0.14	F	1	36039159	TFAP2E	5'UTR	Island
cg26372517	*Homo sapiens*	0.5	0.51	F	1	36039159	TFAP2E	5'UTR	Island
cg26372517	*Macaca mulatta*	0.11	0.11	F	1	36039159	TFAP2E	5'UTR	Island
cg26372517	*Mus musculus*	-0.06	-0.06	F	1	36039159	TFAP2E	5'UTR	Island
cg26372517	*Ovis aries*	-0.16	-0.19	F	1	36039159	TFAP2E	5'UTR	Island
cg26372517	*Rattus norvegicus*	-0.19	-0.12	F	1	36039159	TFAP2E	5'UTR	Island
cg26880891	*Canis lupus familiaris*	0.06	0.03	F	15	72767139	ARIH1	1stExon	Island
cg26880891	*Capreolus capreolus*	-0.31	-0.3	F	15	72767139	ARIH1	1stExon	Island
cg26880891	*Equus caballus*	-0.12	-0.11	F	15	72767139	ARIH1	1stExon	Island
cg26880891	*Felis catus*	-0.17	-0.13	F	15	72767139	ARIH1	1stExon	Island
cg26880891	*Homo sapiens*	-0.44	-0.51	F	15	72767139	ARIH1	1stExon	Island
cg26880891	*Macaca mulatta*	-0.15	-0.15	F	15	72767139	ARIH1	1stExon	Island
cg26880891	*Mus musculus*	0.28	0.33	F	15	72767139	ARIH1	1stExon	Island
cg26880891	*Ovis aries*	0.1	0.18	F	15	72767139	ARIH1	1stExon	Island
cg26880891	*Rattus norvegicus*	-0.36	-0.42	F	15	72767139	ARIH1	1stExon	Island

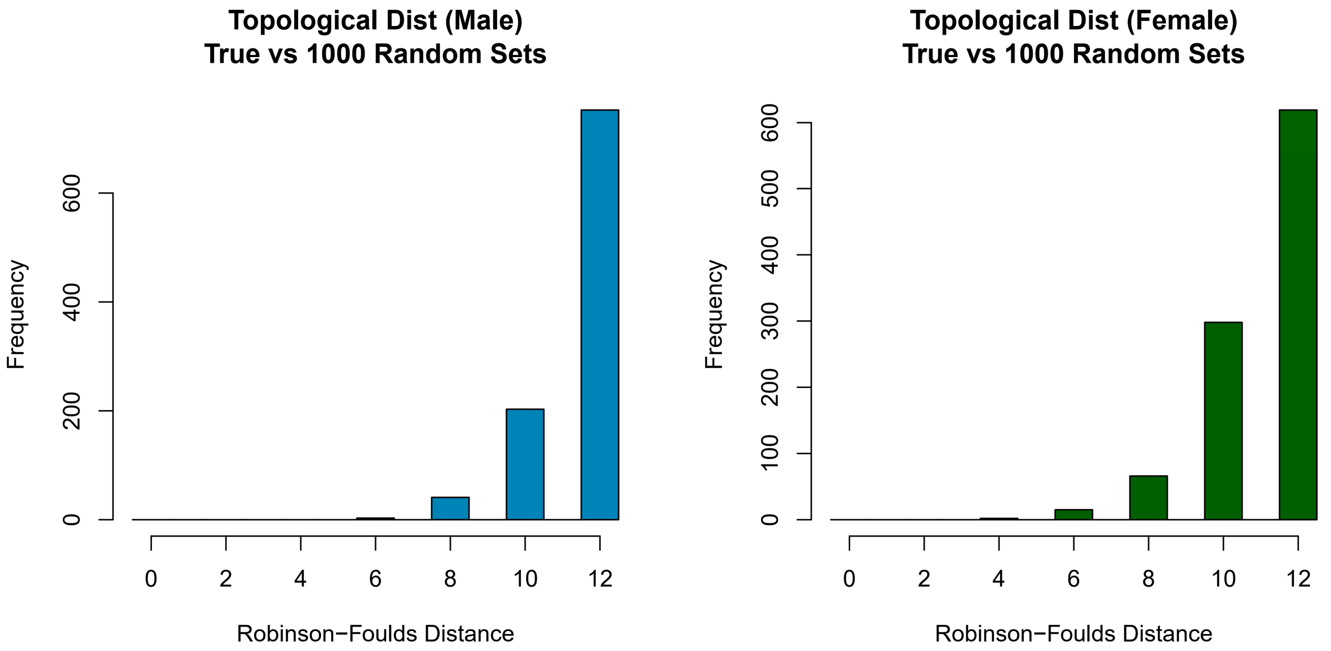

Figure S5. Null-model sensitivity analysis of the epigenetic tree topology. To determine whether the interspecies clustering topology of the 26 OBS-associated CpGs was statistically distinct from random genomic noise, we generated 1,000 random Neighbor-Joining trees. Each random tree was constructed by randomly sampling a size-matched number of CpGs (n=8 for males, n=18 for females) from the entire 37,000 conserved mammalian CpG pool. Histograms display the frequency distribution of Robinson-Foulds (RF) topological distances between these 1,000 random trees and the true OBS-associated CpG tree. The distinct separation from a distance of zero confirms that the clustering pattern driven by the OBS-associated loci represents a specific evolutionary trajectory rather than random background variance.
